# Association of Arterial Hyperoxia With Outcomes in Critically Ill Children: A Systematic Review and Meta-analysis

**DOI:** 10.1001/jamanetworkopen.2021.42105

**Published:** 2022-01-05

**Authors:** Thijs A. Lilien, Nina S. Groeneveld, Faridi van Etten-Jamaludin, Mark J. Peters, Corinne M. P. Buysse, Shawn L. Ralston, Job B. M. van Woensel, Lieuwe D. J. Bos, Reinout A. Bem

**Affiliations:** 1 Pediatric Intensive Care Unit, Emma Children’s Hospital, Amsterdam UMC, Amsterdam, the Netherlands; 2 Research Support, Medical Library AMC, Amsterdam UMC, University of Amsterdam, Amsterdam, the Netherlands; 3 Paediatric Intensive Care, Great Ormond St Hospital and Respiratory, Critical Care and Anesthesia Unit, UCL Great Ormond Street Institute of Child Health, NIHR Biomedical Research Centre, London, United Kingdom; 4 Intensive Care and Department of Pediatric Surgery, Erasmus MC Sophia Children’s Hospital, Rotterdam, the Netherlands; 5 Department of Pediatrics, University of Washington, Seattle; 6 Intensive Care Unit, Amsterdam UMC, Amsterdam, the Netherlands

## Abstract

**Importance:**

Oxygen supplementation is a cornerstone treatment in pediatric critical care. Accumulating evidence suggests that overzealous use of oxygen, leading to hyperoxia, is associated with worse outcomes compared with patients with normoxia.

**Objectives:**

To evaluate the association of arterial hyperoxia with clinical outcome in critically ill children among studies using varied definitions of hyperoxia.

**Data Sources:**

A systematic search of EMBASE, MEDLINE, Cochrane Library, and ClinicalTrials.gov from inception to February 1, 2021, was conducted.

**Study Selection:**

Clinical trials or observational studies of children admitted to the pediatric intensive care unit that examined hyperoxia, by any definition, and described at least 1 outcome of interest. No language restrictions were applied.

**Data Extraction and Synthesis:**

The Meta-analysis of Observational Studies in Epidemiology guideline and Newcastle-Ottawa Scale for study quality assessment were used. The review process was performed independently by 2 reviewers. Data were pooled with a random-effects model.

**Main Outcomes and Measures:**

The primary outcome was 28-day mortality; this time was converted to mortality at the longest follow-up owing to insufficient studies reporting the initial primary outcome. Secondary outcomes included length of stay, ventilator-related outcomes, extracorporeal organ support, and functional performance.

**Results:**

In this systematic review, 16 studies (27 555 patients) were included. All, except 1 randomized clinical pilot trial, were observational cohort studies. Study populations included were post–cardiac arrest (n = 6), traumatic brain injury (n = 1), extracorporeal membrane oxygenation (n = 2), and general critical care (n = 7). Definitions and assessment of hyperoxia differed among included studies. Partial pressure of arterial oxygen was most frequently used to define hyperoxia and mainly by categorical cutoff. In total, 11 studies (23 204 patients) were pooled for meta-analysis. Hyperoxia, by any definition, showed an odds ratio of 1.59 (95% CI, 1.00-2.51; after Hartung-Knapp adjustment, 95% CI, 1.05-2.38) for mortality with substantial between-study heterogeneity (*I*^2^ = 92%). This association was also found in less heterogeneous subsets. A signal of harm was observed at higher thresholds of arterial oxygen levels when grouped by definition of hyperoxia. Secondary outcomes were inadequate for meta-analysis.

**Conclusions and Relevance:**

These results suggest that, despite methodologic limitations of the studies, hyperoxia is associated with mortality in critically ill children. This finding identifies the further need for prospective observational studies and importance to address the clinical implications of hyperoxia in critically ill children.

## Key Points

**Question:**

What is the association between arterial hyperoxia and outcomes in critically ill children admitted to the pediatric intensive care unit?

**Findings:**

This systematic review of 16 studies and meta-analysis of 11 studies comprising a total of 23 204 patients found an association between hyperoxia and mortality in children admitted to the pediatric intensive care unit.

**Meaning:**

These findings suggest that hyperoxia is associated with harm in critically ill children; the clinical implication of this association needs to be addressed in future studies.

## Teaser

his systematic review and meta-analysis assesses outcomes associated with arterial hyperoxia in critically ill children.

## Introduction

The administration of supplemental oxygen is a cornerstone treatment in critically ill patients to prevent and resolve cellular hypoxia. Although oxygen therapy can be lifesaving on many occasions, its overzealous use may lead to supraphysiological levels of the Pao_2_ (ie, hyperoxia), which is associated with deleterious outcomes. For example, in critically ill adults, arterial hyperoxia has been associated with increased mortality, most pronounced at extreme levels of Pao_2_.^1,2^ Detrimental effects of hyperoxia may derive from increased formation of reactive oxygen species, which damage biomolecules, or from its pulmonary and vascular complications, such as atelectasis and hyperoxemic vasoconstriction.^3,4,5,6,7^

In the field of pediatrics, traditionally, most attention on oxygen toxicity has been focused on preterm neonates, a specific population with high vulnerability to develop hyperoxia-associated chronic disease, such as bronchopulmonary dysplasia and retinopathy of prematurity.^8,9^ A growing body of evidence suggests a similar hazard exists for increased mortality and morbidity associated with hyperoxia in critically ill children beyond the direct neonatal period.^10,11^ This increase may be equally important with respect to long-term disease trajectories, as many organs and tissues, including the lungs, continue to develop and mature for several years after birth.^12,13^ Yet, it remains unclear how generalizable these findings are, because these studies address various populations in the pediatric intensive care unit (PICU) and apply different definitions of hyperoxia. Consequently, the current practice of oxygen therapy and target oxygenation levels in the PICU are largely based on expert opinion.^14,15,16^ Rigorous synthesis of the literature on the association between hyperoxia and clinical outcomes in the PICU is necessary to aid in the development of novel study protocols and future strategies to prevent oxygen-associated injury.

In this study, our aims were to describe the definitions of hyperoxia and evaluate the association between hyperoxia and outcomes in critically ill children admitted to the PICU. We hypothesized that hyperoxia was associated with worse outcomes in critically ill children.

## Methods

### Data Sources and Searches

The literature search was performed in consultation with an experienced clinical librarian (F.v.E-J.). We searched the electronic databases Ovid MEDLINE (1946 to February 1, 2021), Ovid EMBASE (1947 to February 1, 2021), Cochrane Central Register of Controlled Trials (from inception to February 1, 2021), and ClinicalTrials.gov. References of full-text articles were screened for relevant studies. No language restrictions were applied. The complete search strategy is reported in eTable 1 in the Supplement. This review is reported in accordance with the Meta-analysis of Observational Studies in Epidemiology (MOOSE) reporting guideline^17^ and the Preferred Reporting Items for Systematic Reviews and Meta-analyses (PRISMA) guidelines.^18^ The protocol of this review was published before start of the study in the PROSPERO International Prospective Register of Systematic Reviews database.^19^

### Study Selection

The citations were screened independently by 2 of us (T.A.L. and N.S.G.) on title and abstract, and discrepancies were resolved through discussion. Full-text articles on potentially eligible studies were retrieved and checked independently for eligibility. Eligible studies were those including patients admitted to the PICU that examined hyperoxia and contained at least 1 outcome of interest. No predefined inclusion criteria were made for the definition of hyperoxia. Only clinical trials and cohort studies were selected. Studies that included only patients with cyanotic congenital heart disease were excluded, as were conference abstracts.

### Outcome Measures

The primary outcome was all-cause 28-day mortality. Secondary outcomes were all-cause mortality at any follow-up, incidence and duration of invasive mechanical ventilation, duration of respiratory support, incidence of acute kidney injury, incidence and duration of organ support (continuous venovenous hemofiltration, extra corporeal membrane oxygenation [ECMO]), PICU and hospital length of stay, long-term lung function (spirometry or forced oscillation technique), and general functional status (Pediatric Overall Performance Category or Pediatric Cerebral Performance Category).^20^

### Data Extraction and Quality Assessment

Data were extracted independently by 2 of us (T.A.L. and N.S.G.) using a structured data extraction form. Differences were resolved through discussion. When necessary, 2 attempts were made to contact authors for missing data or to elucidate uncertainties.

Quality assessment of observational studies remains dubious in comparison with that of randomized clinical trials.^17^ Risk of bias was assessed with the Newcastle-Ottawa scale.^21^ Scoring was performed independently by 2 of us (T.A.L. and N.S.G.) and discrepancies were resolved through discussion with a third reviewer (R.A.B.). As previously suggested, a score below 7 was defined as moderate quality and below 4 as poor.^22^ Randomized clinical trials were scored using the Cochrane Handbook.^23^ Authors were also contacted for individual patient data meta-analysis; however, owing to insufficient response, this analysis was not deemed feasible.

### Statistical Analysis

Studies were grouped by patient population. If populations between studies overlapped, the study with the largest sample size was chosen for analysis. Studies including more than 10% to 15% noneligible patients (eg, adults) were omitted from quantitative analysis. Dichotomous outcomes are reported as odds ratio (OR) and corresponding 95% CI. The reported number of events and total number of events were used to calculate ORs; if only ORs were reported, these values were used. Unadjusted and adjusted ORs were analyzed separately. Inverse variance was used to pool all ORs in a random-effects model. Data were analyzed by the DerSimonian and Laird method, because we expected large differences in sample sizes. To account for the tendency of the DerSimonian and Laird method to result in smaller CIs,^24^ additional Hartung-Knapp adjustment of the pooled effect size 95% CIs was performed.^25^

Heterogeneity was assessed using *I*^2^ statistics. The contribution of studies to the overall heterogeneity was explored by leave-1-out analyses and graphical display of study heterogeneity plots.^26^ Predefined subgroup analyses were performed to analyze more homogenous groups (1) based on outlier analyses, (2) excluding ECMO-only studies, and (3) in studies correcting for confounders.

In addition, studies were grouped by their definition of hyperoxia to determine the association between the design and observed outcomes. Data were pooled for all definitions used or from which data were extractable. Publication bias was assessed with the use of funnel plots to explore small study effect size and *P* curve analysis to explore *P* hacking.^27^

Statistical significance was defined as a 2-sided *P* value <.05. Analyses were performed using RevMan, version 5.3 (The Cochrane Collaboration) and R, version 3.6.1 (R Foundation for Statistical Computing) with RStudio, version 1.2.1335 (RStudio).

## Results

The literature search resulted in 1817 potentially relevant studies. After the screening process, 16 studies, including 27 555 patients, remained (eFigure 1 in the Supplement).^28,29,30,31,32,33,34,35,36,37,38,39,40,41,42,43^ All included studies, except for 1 pilot randomized clinical trial, were observational (12 retrospective and 3 prospective). Study populations included were post–cardiac arrest (n = 6), traumatic brain injury (n = 1), ECMO (n = 2), and general critical care patients (n = 7). Other study characteristics are reported in Table 1. Newcastle-Ottawa scale scores for quality ranged from 7 to 9, with lack of correction for confounders the main contributor to a higher risk of bias (eTable 2 in the Supplement).

**Table 1.** Study Characteristics

Source	Design	Patients, No.	Age, median (IQR)	Population	Main measure of hyperoxia	Definition for hyperoxia	Main outcomes as by design
Bennett et al,^28^ 2013, US	RC	195	Only reported proportions for age groups	Cardiac arrest	Pao_2_	>200 mm Hg (27 kPa)	In-hospital mortality, neurological outcome
Cashen et al,^29^ 2018, US	PC	484	Only reported proportions for age groups	ECMO	Pao_2_	>200 torr (27 kPa)	In-hospital mortality, kidney failure, LOS PICU, LOS hospital, ECMO
Del Castillo et al,^30^ 2012, international (RIBEPCI Network)	PC	223	14 (5-60) mo	Cardiac arrest	Pao_2_ and P/F	>300 mm Hg (40 kPa)	In-hospital mortality
						P/F>300	
Ferguson et al,^31^ 2012, UK	RC	1875	11 (2-61) mo	Cardiac arrest	Pao_2_	>300 mm Hg (40 kPa)	In-hospital mortality
Guerra-Wallace et al,^32^ 2013, US	RC	74	Median (range), 1.8 (0-18) y	Cardiac arrest	Pao_2_	>200 mm Hg (27 kPa)	6-mo mortality
						>300 mm Hg (40 kPa)	
Ketharanathan et al,^33^ 2020, the Netherlands	RC	71	8.9 (4.6-12.9), y	Severe traumatic brain injury	Pao_2_	>200 mm Hg (27 kPa)	In-PICU mortality
						>250 mm Hg (33 kPa)	
						>300 mm Hg (40 kPa)	
Kraft et al,^34^ 2017, Austria	RC	419	Mean (SD), 57.8 (19.9) y	General PICU or ICU	Pao_2_	>120 mm Hg (>16 kPa)	In-hospital mortality, LOS ICU, LOS hospital
López-Herce et al,^35^ 2014, international (Latin America, Spain, Portugal, Italy)	PC	502	44.5 (5-60) mo	Cardiac arrest	Pao_2_ and Fio_2_	≥200 mm Hg (27 kPa)	In-hospital mortality, neurological outcome
						Fio_2_ 0.50-0.79	
						Fio_2_≥0.80	
Numa et al,^36^ 2018, Australia	RC	1447	1.7 (0.3-7.1) y	General PICU (mostly postoperative)	Pao_2_	>250 mm Hg (33 kPa)	In-PICU mortality
Pelletier et al,^37^ 2020, US	RC	4469 or 4537	1.8 (0.4-8.4) y	General PICU	Pao_2_	No predefined cutoff; divided by bands of 100 mm Hg	In-hospital mortality
Peters et al,^38^ 2018, UK	RCT	159	Liberal median, 0.8 (0.1-2.0) y	General PICU	Spo_2_ (target)	Liberal >94%	Feasibility of trial, LOS PICU, IMV duration, 30 VFD, in-PICU mortality
			Conservative median, 1.9 (0.4-5.0) y			Control 88%-92%	
Raman et al,^39^ 2016, UK	RC	7410	Not reported	General PICU	Pao_2_	>300 mm Hg (>40 kPa)	Mortality (unspecified time point)
Ramgopal et al,^40^ 2019, US	RC	6250	Only reported proportions for age groups	General PICU	Pao_2_	≥300 mm Hg (40 kPa)	In-hospital mortality
Ramgopal et al,^41^ 2020, US	RC	3616	Mean (SD), 8.7 (6.7) y	General PICU	Pao_2_	≥300 mm Hg (40 kPa)	In-hospital mortality
Sznycer-Taub et al,^42^ 2016, US	RC	93	7 (5-20) d	ECMO	Pao_2_	>193 mm Hg (26 kPa)	30-d mortality, in-hospital mortality, kidney failure, LOS PICU, LOS hospital
van Zellem et al,^43^ 2015, the Netherlands	RC	200	Nonsurvivors, 20.4 (1.0-211.9) mo	Cardiac arrest	Pao_2_	>200 mm Hg (27 kPa)	In-hospital mortality
						>250 mm Hg (33 kPa)	
			Survivors, 37.6 (1.0-262.6) mo			>300 mm Hg (40 kPa)	

Abbreviations: ECMO, extracorporeal membrane oxygenation; Fio_2_, fraction of inspired oxygen; ICU, intensive care unit; IMV, invasive mechanical ventilation; LOS, length of stay; Pao_2_, arterial partial pressure of oxygen; PC, prospective cohort study; P/F, arterial partial pressure of oxygen to fraction of inspired oxygen ratio; PICU, pediatric intensive care unit; RC, retrospective cohort study; RCT, randomized clinical trial; Spo_2_, peripheral saturation of oxygen; VFD, ventilator-free days.

SI conversion factor: To convert millimeters of mercury to kilopascals, multiply by 0.133.

### Hyperoxia Parameters and Definitions

Definitions, period of assessment, and measures of hyperoxia are reported in Table 2. The Pao_2_ was most frequently used to assess hyperoxia (15 studies) and was usually defined as a categorical predictor with thresholds ranging from greater than 120 mm Hg (to convert to kilopascals, multiply by 0.133) up to greater than 300 mm Hg. In these studies, either absence of hyperoxia (ie, all patients below the threshold) or a custom range for normoxia was used as the comparator. With the custom range approach, the lower limit of normoxia was 60 mm Hg and the threshold for hyperoxia was the upper limit. Most studies evaluated hyperoxia within the first 24 hours of PICU admission. Several measures were used to select Pao_2_, but the highest value and the first value were the most common. Less common methods focused on cumulative exposure by grouping patients by the frequency of Pao_2_ values in the hyperoxic range (1 study^40^) or by analyzing the area under the Pao_2_ to time relationship (3 studies^33,40,43^).

**Table 2.** Overview of Definitions and Assessment Periods of Hyperoxia^a^

Hyperoxia definition	Assessment period		Selection criterion	Source
	Start	End		
**Pao_2_**				
>120 mm Hg (16 kPa)	Start of IMV	IMV day 7	Time-weighted mean	Kraft et al,^34^ 2017
>193 mm Hg (26 kPa)	Start of ECMO	48 h after ECMO initiation	Mean value	Sznycer-Taub et al,^42^ 2016
>200 mm Hg (27 kPa)	At ROSC	1 h after ROSC	None; 1 value	López-Herce et al,^35^ 2014
	24 h after cardiac arrest	24 h after cardiac arrest		
	At ROSC	6 h after ROSC	Highest and lowest value	Bennett et al,^28^ 2013
	PICU admission	24 h after PICU admission	Not specified	Guerra-Wallace et al,^32^ 2013
	PICU admission	24 h after PICU admission	Highest and cumulative exposure	Ketharanathan et al,^33^ 2020
	PICU admission	24 h after PICU admission	Highest and cumulative exposure	van Zellem et al,^43^ 2015
	Start of ECMO	48 h after ECMO initiation	Highest	Cashen et al,^29^ 2018
>250 mm Hg (33 kPa)	PICU admission	1 h after PICU admission	First value	Numa et al,^36^ 2018
	PICU admission	24 h after PICU admission	Highest and cumulative exposure	Ketharanathan et al,^33^ 2020
	PICU admission	24 h after PICU admission	Highest and cumulative exposure	van Zellem et al,^43^ 2015
>300 mm Hg (40 kPa)	At ROSC	1 h after ROSC	None; 1 value	Del Castillo et al,^30^ 2012
	24 h after cardiac arrest	24 h after cardiac arrest		
	PICU admission	1 h after PICU admission	First value	Ferguson et al,^31^ 2012
	PICU admission	1 h after PICU admission	First value	Raman et al,^39^ 2016
	6 h preceding PICU admission	6 h after PICU admission	Highest	Ramgopal et al,^41^ 2020
	PICU admission	24 h after PICU admission	Not specified	Guerra-Wallace et al,^32^ 2013
	PICU admission	24 h after PICU admission	Highest and cumulative exposure	Ketharanathan et al,^33^ 2020
	PICU admission	24 h after PICU admission	Highest and cumulative exposure	van Zellem et al,^43^ 2015
	PICU admission	PICU discharge	Highest and cumulative exposure	Ramgopal et al,^40^ 2019
No threshold defined	PICU admission	72 h after PICU admission	Highest	Pelletier et al,^37^ 2020
**Fio_2_**				
>50%	At ROSC	1 h after ROSC	None; 1 value	López-Herce et al,^35^ 2014
	24 h after cardiac arrest	24 h after cardiac arrest		
**P/F**				
>300	At ROSC	1 h after ROSC	None; 1 value	Del Castillo et al,^30^ 2012
	24 h after cardiac arrest	24 h after cardiac arrest		
**Spo_2_**				
>94%	PICU admission	PICU discharge	NA	Peters et al,^38^ 2018

Abbreviations: ECMO, extracorporeal membrane oxygenation; Fio_2_, fraction of inspired oxygen; IMV, invasive mechanical ventilation; NA, not applicable; P/F, arterial partial pressure of oxygen to fraction of inspired oxygen ratio; PICU, pediatric intensive care unit; ROSC, return of spontaneous circulation; Spo_2_, peripheral saturation of oxygen.

^a^ Assessment period during which hyperoxia was assessed and the criterion used to select assessed measurements for all studies included.

### Outcomes

None of the studies reported the predefined primary outcome of 28-day mortality (eTable 3 in the Supplement) and we therefore converted the primary outcome to mortality at the longest follow-up. Other secondary outcomes could not be pooled owing to selective reporting. eFigure 1 in the Supplement shows studies that were omitted from the quantitative analysis.^30,34,38,41,42^

### Mortality

**Figure 1.**
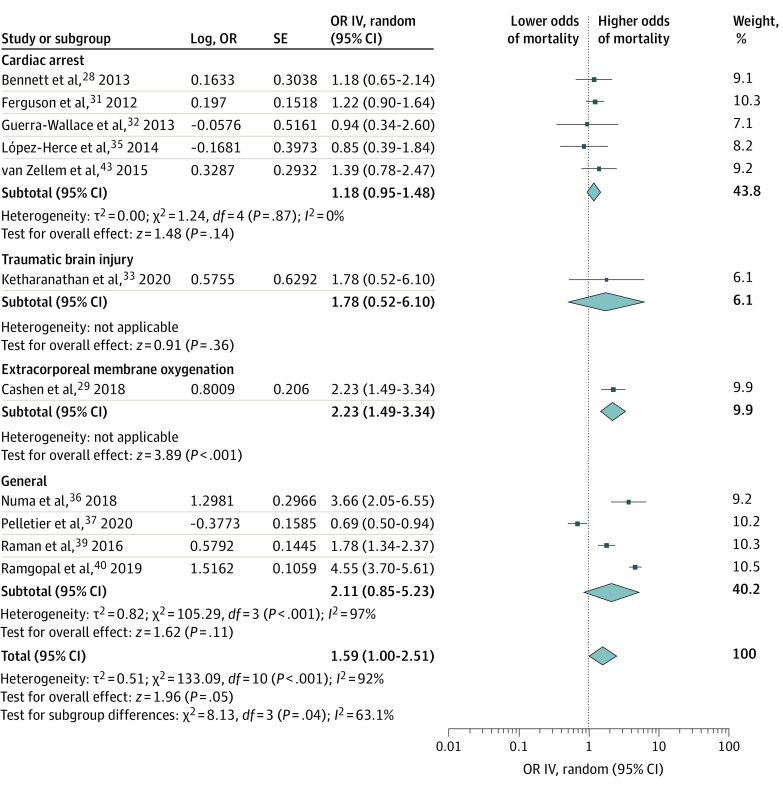
Random-Effects Meta-analysis of Hyperoxia (Categorical Exposure) on Mortality, at Longest Follow-up, Stratified by Case Mix The diamond size represents the summary effect size. IV indicates inverse variance; OR, odds ratio.

The association of hyperoxia with mortality for all 16 studies diverged substantially between studies (eFigure 2 in the Supplement). After omission of the aforementioned investigations,^30,34,38,41,42^ meta-analysis of 11 studies analyzed by subgroup of patients, including 23 204 patients, showed a crude OR of 1.59 (95% CI, 1.00-2.51; *P* = .05) (Figure 1) (95% CI, 1.05-2.38; *P* = .03 after Hartung-Knapp adjustment) (Table 3). Between-study heterogeneity was substantial (*I*^2^ = 92%) and among-subgroup heterogeneity was significant (*I*^2^ = 63%). Heterogeneity was also substantial within the subgroup of general PICU patients (*I*^2^ = 97%). The subgroup of patients receiving ECMO (1 study^29^) showed a significant association (OR, 2.23; 95% CI, 1.49-3.34; *P* < .001).

**Table 3.** Pooled Effect Estimates for Hyperoxia and Mortality^a^

Analysis	Pooled effect estimate, OR (95% CI)	*P* value	95% CI adjusted^b^	*P* value adjusted^b^	*I*^2^, % (95% CI)
Main analysis, all studies in quantitative synthesis	1.59 (1.00-2.51)	.05	1.05-2.38	.03	92 (89-95)
Sensitivity 1, omitting clear outliers^c^	1.58 (1.21-2.07)	<.001	1.14-2.21	.01	58 (12-80)
Sensitivity 2, omitting possible outliers^d^	1.46 (1.17-1.83)	<.001	1.13-1.89	.01	34 (0-71)
Sensitivity 3, omitting ECMO-only studies	1.52 (0.92-2.54)	.11	0.97-2.39	.06	93 (90-96)
Sensitivity 4, confounder-corrected studies	1.51 (0.85-2.68)	.07	0.39-5.85	.32	63 (0-89)

Abbreviations: ECMO, extra corporeal membrane oxygenation; OR, odds ratio.

^a^ Pooled effect estimates from a random-effects model of hyperoxia and mortality using the DerSimonian and Laird method.

^b^ Hartung-Knapp adjustment.

^c^ Omitting Ramgopal et al^40^ and Pelletier et al^37^ based on outlier analyses.

^d^ Omitting Ramgopal et al,^40^ Pelletier et al,^37^ and Numa et al^36^ based on outlier analyses.

We performed several predefined sensitivity analyses to examine the pooled effect size in more homogeneous groups. Outlier analyses indicated 2 pronounced outliers^37,40^ and 1 potential outlier (eFigures 3-5 in the Supplement).^36^ Secondary analyses excluding these outliers showed similar associations to the main analysis and reduced heterogeneity (Table 3; eFigure 6 and eFigure 7 in the Supplement). Secondary analysis, excluding ECMO studies as predefined, resolved among-subgroup heterogeneity, but did not show an association with mortality (Table 3; eFigure 8 in the Supplement).

In addition, we pooled the effect estimate of confounder-adjusted ORs. Twelve studies were corrected for confounders either in multivariable logistic regression or in multivariable nonlinear prediction models.^28,29,31,34,35,36,37,39,40,41,42,43^ Of these, 7 studies corrected for severity of disease using (modified) severity of disease or risk of mortality scores, including Pediatric Logistic Organ Dysfunction, Pediatric Index of Mortality, and Pediatric Risk of Mortality scores.^31,34,36,37,39,40,41^ Adjusted ORs were obtainable from 6 studies and could be pooled for only 3 (Table 3; eFigure 9 in the Supplement).^36,40,43^

**Figure 2.**
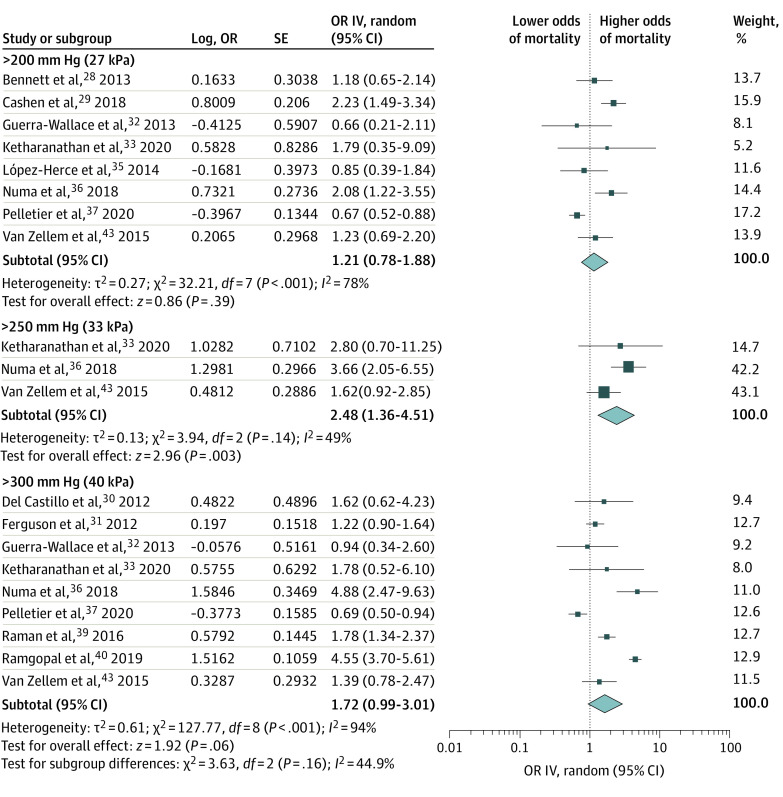
Random-Effects Meta-analysis (Subtotals Only) of Hyperoxia (Categorical Exposure) on Mortality, at Longest Follow-up, Stratified by Threshold of Hyperoxia (Pao_2_) Studies were included for every primary threshold used by the study or from which data were extractable. The diamond size represents the summary effect size. IV indicates inverse variance; OR, odds ratio.

Because we hypothesized that the definition of hyperoxia affects the association with mortality, we pooled study data for each primary threshold that was used or from which data were extractable (Figure 2). Within-group heterogeneity was significant for multiple thresholds (>200 mm Hg: *I*^2^ = 78%; >250 mm Hg: *I*^2^ 49%; >300 mm Hg: *I*^2^ = 94%) and an association between hyperoxia and mortality was observed at the 250 mm Hg threshold (>200 mm Hg: OR, 1.21; 95% CI, 0.78-1.88**;** *P* = .39**;**>250 mm Hg: OR, 2.48; 95% CI, 1.36-4.51**;** *P* = .003; >300 mm Hg: OR, 1.72; 95% CI, 0.99-3.01**;** *P* = .06). Additional sensitivity analyses, based on previous outlier analysis, resolved within-group heterogeneity and showed an association between hyperoxia and mortality at higher thresholds of Pao_2_ (>200 mm Hg: OR, 1.09; 95% CI 0.77-1.53; *P* = .64; *I*^2^ = 0%; >250 mm Hg: OR, 1.75; 95% CI, 1.04-2.95; *P* = .04**;** *I*^2^ = 0%; >300 mm Hg: OR, 1.47; 95% CI, 1.22-1.76; *P* < .001; *I*^2^ = 0%) (eFigure 10 and eFigure 11 in the Supplement)

### Other Outcomes

Other secondary outcomes reported by studies and their outcomes are reported in eTable 3 and eTable 4 in the Supplement. Significant outcomes in terms of incidence and duration of organ support were found in 2 studies including only patients receiving ECMO.^29,42^ One study reported a positive association of hyperoxia with continuous venovenous hemofiltration incidence (hyperoxia vs normoxia: OR, 4.42; 95% CI, 1.19-16.42; *P* = .03), whereas the other study reported a negative association of hyperoxia with ECMO duration (hyperoxia: median, 4.7 [IQR, 2.5-8.0] days vs normoxia: 5.9 [IQR, 3.1-10.5] days; *P* = .009). Associations with hyperoxia were also observed for the length of stay in the pediatric critical care unit^29,34^ and hospital.^34^ Both studies reported an association of hyperoxia with reduced length of stay (length of stay in PICU, hyperoxia: median, 25.0 [IQR, 12.8-48.2] days vs normoxia: median, 30.5 [IQR, 15.6-54.0] days; *P* = .045^29^; hyperoxia: mean [SD], 24.5 [15.9] days vs normoxia: mean [SD], 30.7 [25.2]; *P* = .04^34^; length of stay in hospital, hyperoxia: mean, 43.3 [30.2] days vs normoxia: mean, 58.9 [46.9] days; *P* < .001^34^).

Publication bias was deemed as unlikely, based on the relative symmetrical shape of the funnel plot and exploratory *P* curve results (eFigure 12 and eTable 5 in the Supplement).

## Discussion

In this systematic review, we identified 16 studies including 27 555 patients that described the association between hyperoxia and patient outcomes in critically ill children. Most evidence consisted of observational studies with heterogeneous study designs to define and assess hyperoxia. Five studies were excluded from quantitative synthesis. Meta-analysis of the remaining 11 studies showed, despite substantial heterogeneity, an association between hyperoxia and mortality in the main-analysis and in heterogeneity resolved sensitivity analyses, with a signal of harm at higher thresholds of Pao_2_ when accounted for heterogeneity. Reports on outcomes other than mortality were scarce and inadequate for meta-analysis.

Our results may have been affected by differences in study design, such as the method used for measurement, the definition by cutoff, and the assessment period. First, the most common variable used to measure hyperoxia was Pao_2_. In contrast to adults, a major downside to this method of measure in children is the less common use of arterial lines in the PICU, which could hamper generalizability of our results, because children with arterial lines might be in clinically worse condition compared with those without. Furthermore, although Pao_2_ gives clear information on the systemic burden of oxygen, it does not necessarily inform us on alveolar hyperoxia (ie, the pulmonary burden of oxygen). For example, patients with severe hypoxemia may be exposed to a high fraction of inspired oxygen (Fio_2_), thus a high pulmonary burden of oxygen, without leading to extremes of Pao_2_ due to, for example, alveolar capillary diffusion disturbances or intrapulmonary shunting.^44^ However, alveolar hyperoxia by itself may lead to lung injury.^45^ Hyperoxia should therefore not only be estimated by Pao_2_, because Fio_2_ might be of equal relevance to evaluate the harm of oxygen. Most of the included studies focused only on systemic hyperoxia, defined by Pao_2_, and thus did not sufficiently address the pulmonary burden of oxygen. Future studies on the potential harm of oxygen supplementation should evaluate both the pulmonary and systemic oxygen burden, for example, by assessment of pulmonary biomarkers of hyperoxia-induced injury.^45^

Second, most studies used a categorical definition of hyperoxia by cutoff. Used thresholds are arbitrary and differences therein may affect the observed association as noted herein. Because detrimental effects from oxygen seem to be dose-dependent, observed in an U-shaped association,^37,39^ a sharp cutoff may not reflect this reality well. The degree of hyperoxia, its duration, and large fluctuations in oxygenation may be more important. In addition, the definition of the reference group is of equal importance to the observed outcome, especially if patients with hypoxemia are included.^28,36,40,42,43^ Furthermore, some patient groups may be more susceptible to hyperoxia. For instance, both studies including only patients receiving ECMO support showed an association between hyperoxia and mortality at lower thresholds of hyperoxia.^29,42^

Third, because hyperoxia-induced oxidative stress seems to be time- and dose-dependent, the assessment period of hyperoxia is also of importance.^33,40,46^ Especially when periods exceeding initial admission day are taken into account, the cumulative exposure instead of a single maximum value may be a better representation of the degree of oxygen exposure. In this regard, one study showed that maximum Pao_2_ correlated poorly to moderately with observed cumulative exposure, defined as the area under the Pao_2_ to time relationship, over the first day of admission.^33^

Another factor of importance is the primary outcome of the studies. Within this review, mortality (PICU and hospital) was the most frequently reported outcome among the studies. Although mortality is undoubtedly the most objective and most severe patient outcome, it is relatively uncommon in the PICU (2%-3%), especially compared with the rate in critically ill adults.^47,48^ In addition, next to potential effects on mortality, hyperoxia may have less-definitive, but equally important, long-term detrimental outcomes, because organ development proceeds during childhood and could be hampered by the toxic effects of oxygen on biomolecules.^3,4,5^ However, we found that outcomes related to such potential long-term effects of hyperoxia in critically ill children are lacking in the current evidence. Future studies addressing hyperoxia in the PICU should bring more focus on long-term outcomes, including lung function and general functional scores, as included by the Oxy-PICU trial.^49^

Our findings partly mirror the literature in critically ill adults. The association between arterial hyperoxia and mortality was also observed in 2 meta-analyses of observational studies in adults.^1,2^ However, the association in critically ill adults appears to be more robust as the CI of the association was narrower and the association was maintained in confounder-adjusted analysis. One explanation may be the difference in power, as these adult reviews included more studies, with more than double the number of patients in their quantitative analysis. Another hypothesis could be that children's susceptibility to oxygen toxic effects differs from that of adults owing to age-related differences in vulnerability,^50,51,52^ which, for example, appears to be true for ventilator-induced lung injury.^53,54^ Although there seems to be a sound pathophysiological explanation for the observed association in this review,^55^ further insight of the complex association between oxygen supplementation and outcome from additional prospective pediatric studies is needed.^10,56^ In addition, the clinical implications need to be addressed in clinical trials. On this note, there have been several trials comparing liberal vs restrictive oxygen targets over the past years in critically ill adults.^44,57,58,59,60^ Although a meta-analysis of the earliest trials supported an increased risk for mortality on use of liberal oxygen targets,^57^ these findings were disputed by a recent updated meta-analysis including more large-scale trials.^58^ The most recent trials also did not find a clear harmful effect of liberal oxygenation on outcomes,^44,59,60^ which suggests that the true effect size of hyperoxia is probably much smaller than previously suggested from observational studies and more complex than thought.

### Strengths and Limitations

A strength of this review is the rigorous search, independent screening, and detailed reporting of methods and findings. The observed association was consistent over multiple sensitivity analyses, including less heterogeneous groups and by grouping of hyperoxia definitions.

The study also has limitations. First, all studies included in the quantitative analysis were observational and most were retrospective. Second, many studies did not correct for severity of illness, most probably owing to small sample sizes or lack of significance in univariable analysis. This lack of correction complicated our sensitivity analysis adjusted for severity of illness. We could not resolve this limitation owing to insufficient response from authors for individual patient data meta-analysis. This lack of data is a noteworthy drawback of our study, because severity of illness is an important confounder. Sicker patients may receive more oxygen, which could lead to an overestimation of the effect of oxygen on mortality. Third, our predefined primary outcome was not reported by any included study. We therefore pooled data from mortality at the longest follow-up point, but this pooling may have contributed to the heterogeneity of our results. Fourth, there was substantial heterogeneity between studies, because of both the designs used and within the pooled effect estimates. Although the observed association was consistent in less heterogeneous groups, the wide variation in patient populations, definitions of hyperoxia, and assessment periods warrants caution with generalizability of these results.

## Conclusions

This systematic review and meta-analysis of observational studies suggests an association between hyperoxia and mortality in the PICU, despite methodologic limitations of the included studies. Our findings support that oxygen may be harmful above a certain dose in critically ill children and that it perhaps should be supplied with caution. However, the substantial heterogeneity and the observational design of the included studies warrants judicious interpretation. Future research should further examine the implications of this association and its complex translation into clinical practice as observed in adults.

## Notes

Supplement. **eTable 1.** Search Strategy **eTable 2.** Newcastle-Ottawa-Score for Quality of Cohort Studies **eTable 3.** Overview of All Assessed Outcomes in This Review That Were Reported in Included Studies **eTable 4.** Other Secondary Outcomes Found in Included Studies **eTable 5.** P-Curve Summary of Statistics **eFigure 1.** PRISMA Flowchart of Study Screening Process **eFigure 2.** Forest Plot of Hyperoxia (Categorical) and Mortality, Longest Follow-up, Stratified by Case Mix **eFigure 3.** Bajaut Plot of the Contribution to the Overall Heterogeneity and Pooled Effect Estimate of Each Study **eFigure 4.** Leave-One-Out Plots Sorted by the Effect on Overall Heterogeneity and the Pooled Effect Estimate **eFigure 5.** GOSH Plot of All Subsets of Studies With the Overall Heterogeneity and Estimated Pooled Effect Size and the Three Outlying Studies Based on the GOSH Plot **eFigure 6.** Sensitivity Analysis 1: Excluding Pronounced Outliers **eFigure 7.** Sensitivity Analysis 2: Excluding Pronounced and Potential Outliers **eFigure 8.** Sensitivity Analysis 3: Excluding Studies With Only Patients on ECMO Support **eFigure 9.** Sensitivity Analysis 4: Including Only Studies With Adjusted Odds Ratio for Mortality **eFigure 10.** Random-Effects Meta-analysis of Hyperoxia (Categorical) and Mortality, Longest Follow-up, Stratified by the Threshold of Hyperoxia With Exclusion of Pronounced Outlying Studies and Those Including Only Patients on ECMO Support (Subtotals) **eFigure 11.** Random-Effects Meta-analysis of Hyperoxia (Categorical) and Mortality, Longest Follow-up, Stratified by the Threshold of Hyperoxia With Exclusion of Pronounced and Potential Outlying Studies and Those Including Only Patients on ECMO Support (Subtotals) **eFigure 12.** Funnel Plot of Included Studies in the Main Quantitative Synthesis
eReferences

## References

[1] Helmerhorst HJF, Roos-Blom M-J, van Westerloo DJ, de Jonge E. Association between arterial hyperoxia and outcome in subsets of critical illness: a systematic review, meta-analysis, and meta-regression of cohort studies. Crit Care Med. 2015;43(7):1508-1519. doi:10.1097/CCM.0000000000000998 25855899

[2] Damiani E, Adrario E, Girardis M, . Arterial hyperoxia and mortality in critically ill patients: a systematic review and meta-analysis. Crit Care. 2014;18(6):711. doi:10.1186/s13054-014-0711-x 25532567 PMC4298955

[3] Dizdaroglu M, Jaruga P. Mechanisms of free radical–induced damage to DNA. Free Radic Res. 2012;46(4):382-419. doi:10.3109/10715762.2011.653969 22276778

[4] Rubbo H, Trostchansky A, O’Donnell VB. Peroxynitrite-mediated lipid oxidation and nitration: mechanisms and consequences. Arch Biochem Biophys. 2009;484(2):167-172. doi:10.1016/j.abb.2008.11.007 19022215

[5] Hawkins CL, Davies MJ. Detection, identification, and quantification of oxidative protein modifications. J Biol Chem. 2019;294(51):19683-19708. doi:10.1074/jbc.REV119.006217 31672919 PMC6926449

[6] Rothen HU, Sporre B, Engberg G, Wegenius G, Reber A, Hedenstierna G. Prevention of atelectasis during general anaesthesia. Lancet. 1995;345(8962):1387-1391. doi:10.1016/S0140-6736(95)92595-3 7760608

[7] Watson NA, Beards SC, Altaf N, Kassner A, Jackson A. The effect of hyperoxia on cerebral blood flow: a study in healthy volunteers using magnetic resonance phase-contrast angiography. Eur J Anaesthesiol. 2000;17(3):152-159. doi:10.1097/00003643-200003000-00004 10758463

[8] Thébaud B, Goss KN, Laughon M, . Bronchopulmonary dysplasia. Nat Rev Dis Primers. 2019;5(1):78. doi:10.1038/s41572-019-0127-7 31727986 PMC6986462

[9] Hellström A, Smith LEH, Dammann O. Retinopathy of prematurity. Lancet. 2013;382(9902):1445-1457. doi:10.1016/S0140-6736(13)60178-6 23782686 PMC4389630

[10] Peters MJ. Linking hyperoxia and harm: consequence or merely subsequence? Pediatr Crit Care Med. 2021;22(5):501-503. doi:10.1097/PCC.0000000000002709 33953131

[11] Pelletier JH, Ramgopal S, Horvat CM. Hyperoxemia Is associated with mortality in critically ill children. Front Med (Lausanne). 2021;8:675293. doi:10.3389/fmed.2021.675293 34164417 PMC8215123

[12] Bush A. Lung development and aging. Ann Am Thorac Soc. 2016;13(suppl 5):S438-S446. doi:10.1513/AnnalsATS.201602-112AW 28005431

[13] Agustí A, Noell G, Brugada J, Faner R. Lung function in early adulthood and health in later life: a transgenerational cohort analysis. Lancet Respir Med. 2017;5(12):935-945. doi:10.1016/S2213-2600(17)30434-4 29150410

[14] World Health Organization. Oxygen Therapy for Children: A Manual for Health Workers. World Health Organization; 2016.

[15] Raman S, Ray S, Peters MJ. Survey of oxygen delivery practices in UK paediatric intensive care units. Crit Care Res Pract. 2016;2016:6312970. doi:10.1155/2016/6312970 27516901 PMC4969506

[16] Van de Voorde P, Turner NM, Djakow J, . European Resuscitation Council Guidelines 2021: paediatric life support. Resuscitation. 2021;161:327-387. doi:10.1016/j.resuscitation.2021.02.015 33773830

[17] Stroup DF, Berlin JA, Morton SC, . Meta-analysis of observational studies in epidemiology: a proposal for reporting: Meta-analysis of Observational Studies in Epidemiology (MOOSE) group. JAMA. 2000;283(15):2008-2012. doi:10.1001/jama.283.15.2008 10789670

[18] Moher D, Shamseer L, Clarke M, ; PRISMA-P Group. Preferred reporting items for systematic review and meta-analysis protocols (PRISMA-P) 2015 statement. Syst Rev. 2015;4(1):1. doi:10.1186/2046-4053-4-1 25554246 PMC4320440

[19] Lilien T, Groeneveld N, van Etten-Jamaludin F, et al. Systematic review of the association between hyperoxia and outcome in critically-ill children admitted to the pediatric intensive care unit (CRD42021227732). National Institute for Health Research. PROSPERO International Prospective Register of Systematic Reviews. May 26, 2021. Accessed December 22, 2020. https://www.crd.york.ac.uk/prospero/display_record.php?ID=CRD42021227732

[20] Fiser DH. Assessing the outcome of pediatric intensive care. J Pediatr. 1992;121(1):68-74. doi:10.1016/S0022-3476(05)82544-2 1625096

[21] Wells G, Shea B, O’Connell D, The Newcastle-Ottawa Scale (NOS) for assessing the quality of nonrandomised studies in meta-analyses. 2021. Accessed March 26, 2021. http://www.ohri.ca/programs/clinical_epidemiology/oxford.asp

[22] Alobaidi R, Morgan C, Basu RK, . Association between fluid balance and outcomes in critically ill children: a systematic review and meta-analysis. JAMA Pediatr. 2018;172(3):257-268. doi:10.1001/jamapediatrics.2017.4540 29356810 PMC5885847

[23] Higgins J, Thomas J, Chandler J, , eds. Cochrane Handbook for Systematic Reviews of Interventions. version 6.0. July 2019. Accessed March 26, 2021. http://www.training.cochrane.org/handbook

[24] Langan D, Higgins JPT, Jackson D, . A comparison of heterogeneity variance estimators in simulated random-effects meta-analyses. Res Synth Methods. 2019;10(1):83-98. doi:10.1002/jrsm.1316 30067315

[25] Knapp G, Hartung J. Improved tests for a random effects meta-regression with a single covariate. Stat Med. 2003;22(17):2693-2710. doi:10.1002/sim.1482 12939780

[26] Olkin I, Dahabreh IJ, Trikalinos TA. GOSH—a graphical display of study heterogeneity. Res Synth Methods. 2012;3(3):214-223. doi:10.1002/jrsm.1053 26062164

[27] Simonsohn U, Nelson LD, Simmons JP. p-Curve and effect size: correcting for publication bias using only significant results. Perspect Psychol Sci. 2014;9(6):666-681. doi:10.1177/1745691614553988 26186117

[28] Bennett KS, Clark AE, Meert KL, ; Pediatric Emergency Care Medicine Applied Research Network. Early oxygenation and ventilation measurements after pediatric cardiac arrest: lack of association with outcome. Crit Care Med. 2013;41(6):1534-1542. doi:10.1097/CCM.0b013e318287f54c 23552509 PMC3683244

[29] Cashen K, Reeder R, Dalton HJ, ; Eunice Kennedy Shriver National Institute of Child Health and Human Development Collaborative Pediatric Critical Care Research Network (CPCCRN). Hyperoxia and hypocapnia during pediatric extracorporeal membrane oxygenation: associations with complications, mortality, and functional status among survivors. Pediatr Crit Care Med. 2018;19(3):245-253. doi:10.1097/PCC.0000000000001439 29319634 PMC5834382

[30] Del Castillo J, López-Herce J, Matamoros M, ; Iberoamerican Pediatric Cardiac Arrest Study Network RIBEPCI. Hyperoxia, hypocapnia and hypercapnia as outcome factors after cardiac arrest in children. Resuscitation. 2012;83(12):1456-1461. doi:10.1016/j.resuscitation.2012.07.019 22841610

[31] Ferguson LP, Durward A, Tibby SM. Relationship between arterial partial oxygen pressure after resuscitation from cardiac arrest and mortality in children. Circulation. 2012;126(3):335-342. doi:10.1161/CIRCULATIONAHA.111.085100 22723307

[32] Guerra-Wallace MM, Casey FL III, Bell MJ, Fink EL, Hickey RW. Hyperoxia and hypoxia in children resuscitated from cardiac arrest. Pediatr Crit Care Med. 2013;14(3):e143-e148. doi:10.1097/PCC.0b013e3182720440 23392367 PMC3654405

[33] Ketharanathan N, De Jonge RCJ, Klouwen I, . Hyperoxia in pediatric severe traumatic brain injury (TBI): a comparison of patient classification by cutoff versus cumulative (area-under-the-curve) analysis. Brain Inj. 2020;34(7):958-964. doi:10.1080/02699052.2020.1765021 32485120

[34] Kraft F, Andel H, Gamper J, Markstaller K, Ullrich R, Klein KU. Incidence of hyperoxia and related in-hospital mortality in critically ill patients: a retrospective data analysis. Acta Anaesthesiol Scand. 2018;62(3):347-356. doi:10.1111/aas.13047 29210062

[35] López-Herce J, del Castillo J, Matamoros M, ; Iberoamerican Pediatric Cardiac Arrest Study Network RIBEPCI. Post return of spontaneous circulation factors associated with mortality in pediatric in-hospital cardiac arrest: a prospective multicenter multinational observational study. Crit Care. 2014;18(6):607. doi:10.1186/s13054-014-0607-9 25672247 PMC4245792

[36] Numa A, Aneja H, Awad J, . Admission hyperoxia is a risk factor for mortality in pediatric intensive care. Pediatr Crit Care Med. 2018;19(8):699-704. doi:10.1097/PCC.0000000000001630 29927878

[37] Pelletier JH, Ramgopal S, Au AK, Clark RSB, Horvat CM. Maximum Pao_2_ in the first 72 hours of intensive care is associated with risk-adjusted mortality in pediatric patients undergoing mechanical ventilation. Crit Care Explor. 2020;2(9):e0186. doi:10.1097/CCE.0000000000000186 32984827 PMC7491884

[38] Peters MJ, Jones GAL, Wiley D, ; Oxy-PICU Investigators for the Paediatric Intensive Care Society Study Group (PICS-SG). Conservative versus liberal oxygenation targets in critically ill children: the randomised multiple-centre pilot Oxy-PICU trial. Intensive Care Med. 2018;44(8):1240-1248. doi:10.1007/s00134-018-5232-7 29868973

[39] Raman S, Prince NJ, Hoskote A, Ray S, Peters MJ. Admission Pao_2_ and mortality in critically ill children: a cohort study and systematic review. Pediatr Crit Care Med. 2016;17(10):e444-e450. doi:10.1097/PCC.0000000000000905 27509363

[40] Ramgopal S, Dezfulian C, Hickey RW, . Association of severe hyperoxemia events and mortality among patients admitted to a pediatric intensive care unit. JAMA Netw Open. 2019;2(8):e199812. doi:10.1001/jamanetworkopen.2019.9812 31433484 PMC6707098

[41] Ramgopal S, Dezfulian C, Hickey RW, . Early hyperoxemia and outcome among critically ill children. Pediatr Crit Care Med. 2020;21(2):e129-e132. doi:10.1097/PCC.0000000000002203 31821205 PMC7304556

[42] Sznycer-Taub NR, Lowery R, Yu S, Owens ST, Hirsch-Romano JC, Owens GE. Hyperoxia is associated with poor outcomes in pediatric cardiac patients supported on venoarterial extracorporeal membrane oxygenation. Pediatr Crit Care Med. 2016;17(4):350-358. doi:10.1097/PCC.0000000000000655 27043897

[43] van Zellem L, de Jonge R, van Rosmalen J, Reiss I, Tibboel D, Buysse C. High cumulative oxygen levels are associated with improved survival of children treated with mild therapeutic hypothermia after cardiac arrest. Resuscitation. 2015;90:150-157. doi:10.1016/j.resuscitation.2014.12.013 25576438

[44] Schjørring OL, Klitgaard TL, Perner A, ; HOT-ICU Investigators. Lower or higher oxygenation targets for acute hypoxemic respiratory failure. N Engl J Med. 2021;384(14):1301-1311. doi:10.1056/NEJMoa2032510 33471452

[45] Cronin WA, Forbes AS, Wagner KL, . Exhaled volatile organic compounds precedes pulmonary injury in a swine pulmonary oxygen toxicity model. Front Physiol. 2019;10:1297. doi:10.3389/fphys.2019.01297 31849689 PMC6901787

[46] Helmerhorst HJF, Schouten LRA, Wagenaar GTM, . Hyperoxia provokes a time- and dose-dependent inflammatory response in mechanically ventilated mice, irrespective of tidal volumes. Intensive Care Med Exp. 2017;5(1):27. doi:10.1186/s40635-017-0142-5 28550659 PMC5446430

[47] Burns JP, Sellers DE, Meyer EC, Lewis-Newby M, Truog RD. Epidemiology of death in the PICU at five US teaching hospitals. Crit Care Med. 2014;42(9):2101-2108. doi:10.1097/CCM.0000000000000498 24979486 PMC4134743

[48] Capuzzo M, Volta C, Tassinati T, ; Working Group on Health Economics of the European Society of Intensive Care Medicine. Hospital mortality of adults admitted to intensive care units in hospitals with and without intermediate care units: a multicentre European cohort study. Crit Care. 2014;18(5):551. doi:10.1186/s13054-014-0551-8 25664865 PMC4261690

[49] Registry ISRCTN. A randomised multiple centre trial of conservative versus liberal oxygenation targets in critically ill children (Oxy-PICU) (ISRCTN92103439). July 20, 2020. Accessed November 30, 2021. https://www.isrctn.com/ISRCTN92103439

[50] Yam J, Frank L, Roberts RJ. Oxygen toxicity: comparison of lung biochemical responses in neonatal and adult rats. Pediatr Res. 1978;12(2):115-119. doi:10.1203/00006450-197802000-00010 643379

[51] Stevens JB, Autor AP. Induction of superoxide dismutase by oxygen in neonatal rat lung. J Biol Chem. 1977;252(10):3509-3514. doi:10.1016/S0021-9258(17)40420-0 863894

[52] Autor AP, Frank L, Roberts RJ. Developmental characteristics of pulmonary superoxide dismutase: relationship to idiopathic respiratory distress syndrome. Pediatr Res. 1976;10(3):154-158. doi:10.1203/00006450-197603000-00002 1250644

[53] Kneyber MCJ, Zhang H, Slutsky AS. Ventilator-induced lung injury: similarity and differences between children and adults. Am J Respir Crit Care Med. 2014;190(3):258-265. doi:10.1164/rccm.201401-0168CP 25003705 PMC4896812

[54] Schouten LRA, Schultz MJ, van Kaam AH, Juffermans NP, Bos AP, Wösten-van Asperen RM. Association between maturation and aging and pulmonary responses in animal models of lung injury: a systematic review. Anesthesiology. 2015;123(2):389-408. doi:10.1097/ALN.0000000000000687 25919403

[55] Helmerhorst HJF, Schultz MJ, van der Voort PHJ, de Jonge E, van Westerloo DJ. Bench-to-bedside review: the effects of hyperoxia during critical illness. Crit Care. 2015;19(1):284. doi:10.1186/s13054-015-0996-4 26278383 PMC4538738

[56] Horvat C. Statistical note: confounding and causality in observational studies. Pediatr Crit Care Med. 2021;22(5):496-498. doi:10.1097/PCC.0000000000002702 33721879 PMC8882362

[57] Chu DK, Kim LHY, Young PJ, . Mortality and morbidity in acutely ill adults treated with liberal versus conservative oxygen therapy (IOTA): a systematic review and meta-analysis. Lancet. 2018;391(10131):1693-1705. doi:10.1016/S0140-6736(18)30479-3 29726345

[58] Barbateskovic M, Schjørring OL, Krauss SR, . Higher vs lower oxygenation strategies in acutely ill adults: a systematic review with meta-analysis and trial sequential analysis. Chest. 2021;159(1):154-173. doi:10.1016/j.chest.2020.07.015 32687907

[59] Gelissen H, de Grooth HJ, Smulders Y, . Effect of low-normal vs high-normal oxygenation targets on organ dysfunction in critically ill patients: a randomized clinical trial. JAMA. 2021;326(10):940-948. doi:10.1001/jama.2021.13011 34463696 PMC8408761

[60] Barrot L, Asfar P, Mauny F, ; LOCO2 Investigators and REVA Research Network. Liberal or conservative oxygen therapy for acute respiratory distress syndrome. N Engl J Med. 2020;382(11):999-1008. doi:10.1056/NEJMoa1916431 32160661

