# Supplementary material for: Association of Arterial Hyperoxia With Outcomes in Critically Ill Children: A Systematic Review and Meta-analysis
Source: JAMA Netw Open. 2022 Jan 5;5(1):e2142105. doi: 10.1001/jamanetworkopen.2021.42105 (PMC8733830; doi:10.1001/jamanetworkopen.2021.42105)
Supplement: Supplement. — eTable 1. Search Strategy eTable 2. Newcastle-Ottawa-Score for Quality of Cohort Studies eTable 3. Overview of All Assessed Outcomes in This Review That Were Reported in Included Studies eTable 4. Other Secondary Outcomes Found in Included Studies eTable 5. P-Curve Summary of Statistics eFigure 1. PRISMA Flowchart of Study Screening Process eFigure 2. Forest Plot of Hyperoxia (Categorical) and Mortality, Longest Follow-up, Stratified by Case Mix eFigure 3. Bajaut Plot of the Contribution to the Overall Heterogeneity and Pooled Effect Estimate of Each Study eFigure 4. Leave-One-Out Plots Sorted by the Effect on Overall Heterogeneity and the Pooled Effect Estimate eFigure 5. GOSH Plot of All Subsets of Studies With the Overall Heterogeneity and Estimated Pooled Effect Size and the Three Outlying Studies Based on the GOSH Plot eFigure 6. Sensitivity Analysis 1: Excluding Pronounced Outliers eFigure 7. Sensitivity Analysis 2: Excluding Pronounced and Potential Outliers eFigure 8. Sensitivity Analysis 3: Excluding Studies With Only Patients on ECMO Support eFigure 9. Sensitivity Analysis 4: Including Only Studies With Adjusted Odds Ratio for Mortality eFigure 10. Random-Effects Meta-analysis of Hyperoxia (Categorical) and Mortality, Longest Follow-up, Stratified by the Threshold of Hyperoxia With Exclusion of Pronounced Outlying Studies and Those Including Only Patients on ECMO Support (Subtotals) eFigure 11. Random-Effects Meta-analysis of Hyperoxia (Categorical) and Mortality, Longest Follow-up, Stratified by the Threshold of Hyperoxia With Exclusion of Pronounced and Potential Outlying Studies and Those Including Only Patients on ECMO Support (Subtotals) eFigure 12. Funnel Plot of Included Studies in the Main Quantitative Synthesis eReferences [file jamanetwopen-e2142105-s001.pdf]

Supplementary Online Content

Lilien TA, Groeneveld NS, van Etten-Jamaludin F, et al. Association of arterial hyperoxia with outcomes in critically ill children: a systematic review and meta-analysis. *JAMA Netw Open*. 2022;5(1):e2142105. doi:10.1001/jamanetworkopen.2021.42105

eTable 1. Search Strategy

eTable 2. Newcastle-Ottawa-Score for Quality of Cohort Studies

eTable 3. Overview of All Assessed Outcomes in This Review That Were Reported in Included Studies

eTable 4. Other Secondary Outcomes Found in Included Studies

eTable 5. P-Curve Summary of Statistics

eFigure 1. PRISMA Flowchart of Study Screening Process

eFigure 2. Forest Plot of Hyperoxia (Categorical) and Mortality, Longest Follow-up, Stratified by Case Mix

eFigure 3. Bajaut Plot of the Contribution to the Overall Heterogeneity and Pooled Effect Estimate of Each Study

eFigure 4. Leave-One-Out Plots Sorted by the Effect on Overall Heterogeneity and the Pooled Effect Estimate

eFigure 5. GOSH Plot of All Subsets of Studies With the Overall Heterogeneity and Estimated Pooled Effect Size and the Three Outlying Studies Based on the GOSH Plot

eFigure 6. Sensitivity Analysis 1: Excluding Pronounced Outliers

eFigure 7. Sensitivity Analysis 2: Excluding Pronounced and Potential Outliers

eFigure 8. Sensitivity Analysis 3: Excluding Studies With Only Patients on ECMO Support

eFigure 9. Sensitivity Analysis 4: Including Only Studies With Adjusted Odds Ratio for Mortality

eFigure 10. Random-Effects Meta-analysis of Hyperoxia (Categorical) and Mortality, Longest Follow-up, Stratified by the Threshold of Hyperoxia With Exclusion of Pronounced Outlying Studies and Those Including Only Patients on ECMO Support (Subtotals)

eFigure 11. Random-Effects Meta-analysis of Hyperoxia (Categorical) and Mortality, Longest Follow-up, Stratified by the Threshold of Hyperoxia With Exclusion of Pronounced and Potential Outlying Studies and Those Including Only Patients on ECMO Support (Subtotals)

eFigure 12. Funnel Plot of Included Studies in the Main Quantitative Synthesis

eReferences

This supplementary material has been provided by the authors to give readers additional information about their work.

© 2022 Lilien TA et al. *JAMA Network Open.*

eTable 1. Search strategy

**Database**	**MEDLINE (OVID)**	
**Searched from**	**1946 to February 01, 2021**	
#	**Searches**	**Results**
1	Intensive Care Units, Pediatric/	8127
2	(Critical Illness/ or Critical Care/) and (exp Child/ or Pediatrics/)	7984
3	(PICU or PICUs or pediatric ICU* or paediatric ICU* or pediatric intensive care or paediatric intensive care).ti,ab,kf,kw.	11908
4	(critical* adj3 child*).ti,ab,kw,kf.	5910
5	1 or 2 or 3 or 4	22680
6	Hyperoxia/	3829
7	(hyperoxi* or hyperoxemi* or hyperoxaemi* or PAO2 or PO2 or FIO2).ti,ab,kw,kf.	38728
8	((high or increased or elevated or supraphysiol*) adj3 oxygen adj3 (concentra* or tension* or pressure* or level*)).ti,ab,kw,kf.	5801
9	(oxygen adj3 (supplement* or therap* or suppl* or administra* or inspir*)).ti,ab,kw,kf.	35694
10	(arteria* adj3 oxygen adj3 (concentra* or tension* or pressure* or level*)).ti,ab,kw,kf.	6743
11	6 or 7 or 8 or 9 or 10	77518
12	5 and 11	621
**Database**	**EMBASE (OVID)**	
**Searched from**	**1947 to February 01, 2021**	
#	**Searches**	**Results**
1	pediatric intensive care unit/	7209
2	(critically ill patient/ or critical illness/ or intensive care/) and (exp child/ or pediatrics/)	25652
3	(PICU or PICUs or pediatric ICU* or paediatric ICU* or pediatric intensive care or paediatric intensive care).ti,ab,kw.	21148
4	(critical* adj3 child*).ti,ab,kw.	8673
5	1 or 2 or 3 or 4	44904
6	hyperoxia/	10441
7	(hyperoxi* or hyperoxemi* or hyperoxaemi* or PAO2 or PO2 or FIO2).ti,ab,kw.	60208
8	((high or increased or elevated or supraphysiol*) adj3 oxygen adj3 (concentra* or tension* or pressure* or level*)).ti,ab,kw.	7937
9	(oxygen adj3 (supplement* or therap* or suppl* or administra* or inspir*)).ti,ab,kw.	51850
10	(arteria* adj3 oxygen adj3 (concentra* or tension* or pressure* or level*)).ti,ab,kw.	9189
11	6 or 7 or 8 or 9 or 10	115973
12	5 and 11	1505
**Database**	**Cochrane Central Register of Controlled Trials**	
**Searched from**	**Inception to February 2021**	
#	**Searches**	**Results**
1	(PICU or PICUs or pediatric ICU* or paediatric ICU* or pediatric intensive care or paediatric intensive care):ti,ab,kw	2601
2	MeSH descriptor: [Intensive Care Units, Pediatric] explode all trees	1054
3	#1 or #2	3322
4	MeSH descriptor: [Critical Illness] explode all trees	2303
5	MeSH descriptor: [Critical Care] this term only	1738
6	MeSH descriptor: [Child] explode all trees	56347
7	(#4 or #5) and #6	226
8	#3 or #7	3396

© 2022 Lilien TA et al. *JAMA Network Open.*

#	**Searches**	**Results**
9	MeSH descriptor: [Hyperoxia] explode all trees	204
10	(hyperoxi* or hyperoxemi* or hyperoxaemi* or PAO2 or PO2 or FIO2):ti,ab,kw	5963
11	((high or increased or elevated or supraphysiol*) near/3 oxygen near/3 (concentra* or tension* or pressure* or level*)):ti,ab,kw	397
12	(oxygen near/3 (supplement* or therap* or suppl* or administra* or inspir*)):ti,ab,kw	9619
13	(arteria* near/3 oxygen near/3 (concentra* or tension* or pressure* or level*)):ti,ab,kw	2261
14	#9 or #10 or #11 or #12 or #13	15171
15	#8 and #14 in Trials	338

© 2022 Lilien TA et al. *JAMA Network Open.*

eTable 2. Newcastle-Ottawa-Score for quality of cohort studies.

	Selection				Comparability	Outcome			
**Study**	**Representativeness of cohort**	**Selection of non-exposed cohort**	**Ascertainment of exposure**	**Outcome of interest**	**Comparability of cohorts**	**Assessment of outcome**	**Adequate duration of follow-up**	**Adequate follow-up of cohort**	**Total score**
Bennett et al,[1] 2013	A*	A*	A*	A*	B*	B*	A*	A*	8
Cashen et al,[2] 2018	B*	A*	A*	A*	B*	A*	A*	B*	8
Del Castillo et al,[3] 2012	B*	A*	A*	A*	-	A*	A*	B*	7
Ferguson et al,[4] 2012	A*	A*	A*	A*	A* B*	A*	A*	A*	9
Guerra-Wallace et al,[5] 2013	A*	A*	A*	A*	-	A*	A*	A*	7
Ketharanathan et al,[6] 2020	A*	A*	A*	A*	-	A*	A*	A*	7
Kraft et al,[7] 2017	B*	A*	A*	A*	A*B*	A*	A*	B*	9
López-Herce et al,[8] 2014	A*	A*	A*	A*	-	A*	A*	A*	7
Numa et al,[9] 2018	A*	A*	A*	A*	A*	A*	A*	A*	8
Pelletier et al,[10] 2020	A*	A*	A*	A*	A*	A*	A*	A*	8
Peters et al,[11] 2018	NA	NA	NA	NA	NA	NA	NA	NA	NA
Raman et al,[12] 2016	B*	A*	A*	A*	A*B*	A*	A*	A*	9
Ramgopal et al,[13] 2019	A*	A*	A*	A*	A*	A*	A*	A*	8

© 2022 Lilien TA et al. *JAMA Network Open.*

Study	Representativeness of cohort	Selection of non-exposed cohort	Ascertainment of exposure	Outcome of interest	Comparability of cohorts	Assessment of outcome	Adequate duration of follow-up	Adequate follow-up of cohort	Total score
Ramgopal et al,[14] 2020	A*	A*	A*	A*	A*	A*	A*	A*	8
Sznycer-Taub et al,[15] 2016	A*	A*	A*	A*	-	A*	A*	A*	7
Van Zellem et al,[16] 2015	A*	A*	A*	A*	B*	A*	A*	A*	8

Comparability of cohorts: A for severity of disease score, B for age.

© 2022 Lilien TA et al. *JAMA Network Open.*

eTable 3. Overview of all assessed outcomes in this review that were reported in included studies.

Study / Outcome	28-day mortality	In-PICU mortality	In-hospital mortality	6-month mortality	Incidence of IMV	Duration of IMV	Duration of respiratory support	Incidence of AKI	Incidence of organ support	Duration of organ support	Length of stay PICU	Length of stay hospital	Neurological outcome (PCPC)	Long-term functional score	Long-term lung function
Bennett et al,[1] 2013			■		Not reported by any study		Not reported by any study		■		■	■	■	Not reported by any study	Not reported by any study
Cashen et al,[2] 2018			■					■		■	■	■			
Del Castillo et al,[3] 2012			■												
Ferguson et al,[4] 2012			■					■	■						
Guerra-Wallace et al,[5] 2013				■											
Ketharanathan et al,[6] 2020		■													
Kraft et al,[7] 2017			■								■	■			
López-Herce et al,[8] 2014			■										■		
Numa et al,[9] 2018		■													
Pelletier et al,[10] 2020			■												
Peters et al,[11] 2018		■				■				■	■				
Raman et al,[12] 2016	a	a	a	a											
Ramgopal et al,[13] 2019			■												
Ramgopal et al,[14] 2020			■												
Sznycer-Taub et al,[15] 2016	■ b		■						■		■	■			
Van Zellem et al,[16] 2015			■												

eTable 3. Organ support was defined as extra corporeal membrane oxygenation or continuous venovenous hemodialysis. a, Raman et al did not specify at what time point mortality was assessed; b, Sznycer-Taub et al.[15] assessed mortality at day 30 and not precisely at day 28; PICU, pediatric intensive care unit; IMV, invasive mechanical ventilation; AKI, acute kidney injury; PCPC, pediatric cerebral performance category.

© 2022 Lilien TA et al. *JAMA Network Open.*

eTable 4. Other secondary outcomes found in included studies.

Outcome	Hyperoxia (Liberal)[a]	Normoxia (Conservative)[a]	Odds Ratio (95% CI)	*P*-value
**Duration of IMV (days)**				
Peters et al,[11] 2018; median [IQR]	3 [2-6]	3 [2-6]	NA	1.0
**Incidence of AKI**				
Cashen et al,[2] 2018; N (Total)	117 (331)	54 (153)	1.00 (0.67-1.50)	0.99
Ferguson et al,[4] 2012; N (Total)	16 (207)	93 (1220)	1.02 (0.58-1.76)	0.96
**Incidence of organ support**				
Bennet et al,[1] 2013; N (Total)	11 (87)	4 (66)	2.24 (0.68-7.39)	0.184
Ferguson et al,[4] 2012; N (Total)	9 (207)	36 (1220)	1.49 (0.71-3.15)	0.29
Sznycer-Taub et al,[15] 2016; N (Total)	32 (73)	3 (20)	4.42 (1.19-16.42)	**0.026**
**Duration of organ support (days)**				
Cashen et al,[2] 2018; median [IQR]	4.7 [2.5-8.0]	5.9 [3.1-10.5]	NA	**0.009**
Peters et al,[11] 2018; median [IQR]	0 [0]	0 [0]	NA	NA
**Length of stay PICU (days)**				
Bennet et al,[1] 2013; median [IQR]	5 [1-16]	6 [2-12.5]	NA	NA
Cashen et al,[2] 2018; median [IQR]	25.0 [12.8-48.2]	30.5 [15.6-54.0]	NA	**0.045**
Kraft et al,[7] 2017; mean ±SD	24.5 ±15.9	30.7 ±25.2	NA	**0.039**
Peters et al,[11] 2018; median [IQR]	6 [4-11]	5 [4-8]	NA	0.292
Sznycer-Taub et al,[15] 2016; median [IQR]	21 [14-42]	28.5 [20-52]	NA	0.08
**Length of stay hospital (days)**				
Bennet et al,[1] 2013; median [IQR]	9 [1-29]	7 [3-17]	NA	NA
Cashen et al,[2] 2018; median [IQR]	33.2 [13.4-67.5]	39.1 [19.7-64.8]	NA	0.231
Kraft et al,[7] 2017; mean ±SD	43.3 ±30.2	58.9 ±46.9	NA	**0.0003**
Sznycer-Taub et al,[15] 2016; median [IQR]	28 [18-63]	48 [31-64]	NA	0.09
**Neurological outcome (PCPC)**				
Bennett et al,[1] 2013; ≤2[b] N (Total)	30 (87)	37 (88)	1.02 (0.46-2.27)[c]	0.96

© 2022 Lilien TA et al. *JAMA Network Open.*

Outcome	Hyperoxia (Liberal)[a]	Normoxia (Conservative)[a]	Odds Ratio (95% CI)	*P*-value
**Neurological outcome (PCPC)**				
López-Herce et al,[8] 2014; >2 %	16.7[d]	33.3[d]	0.400 (0.103-1.553)	0.186

eTable 4. a, Liberal or Conservative oxygenation applies to Peters et al[11]; b, Or no change if pre-admission PCPC was above 2; c, Adjusted odds ratio; d, PaO2 1h after cardiac arrest. PICU, pediatric intensive care unit; CI, confidence interval; IQR, interquartile range; NA, not available; SD, standard deviation; IMV, invasive mechanical ventilation; AKI, acute kidney injury; PCPC, pediatric cognitive performance category.

© 2022 Lilien TA et al. *JAMA Network Open.*

eTable 5. *P* curve summary of statistics.

		Full Curve		Half Curve		Evidential Value	
	$P_{Binomial}$	z_{Full}	P_{Full}	z_{Half}	P_{Half}	Present	Absent
Right-Skewness Test	0.125	-5.424	0	-5.059	0	Yes	No
Flatness Test	1.000	3.728	1	4.231	1		

© 2022 Lilien TA et al. *JAMA Network Open.*

eFigure 1. PRISMA Flowchart of study screening process

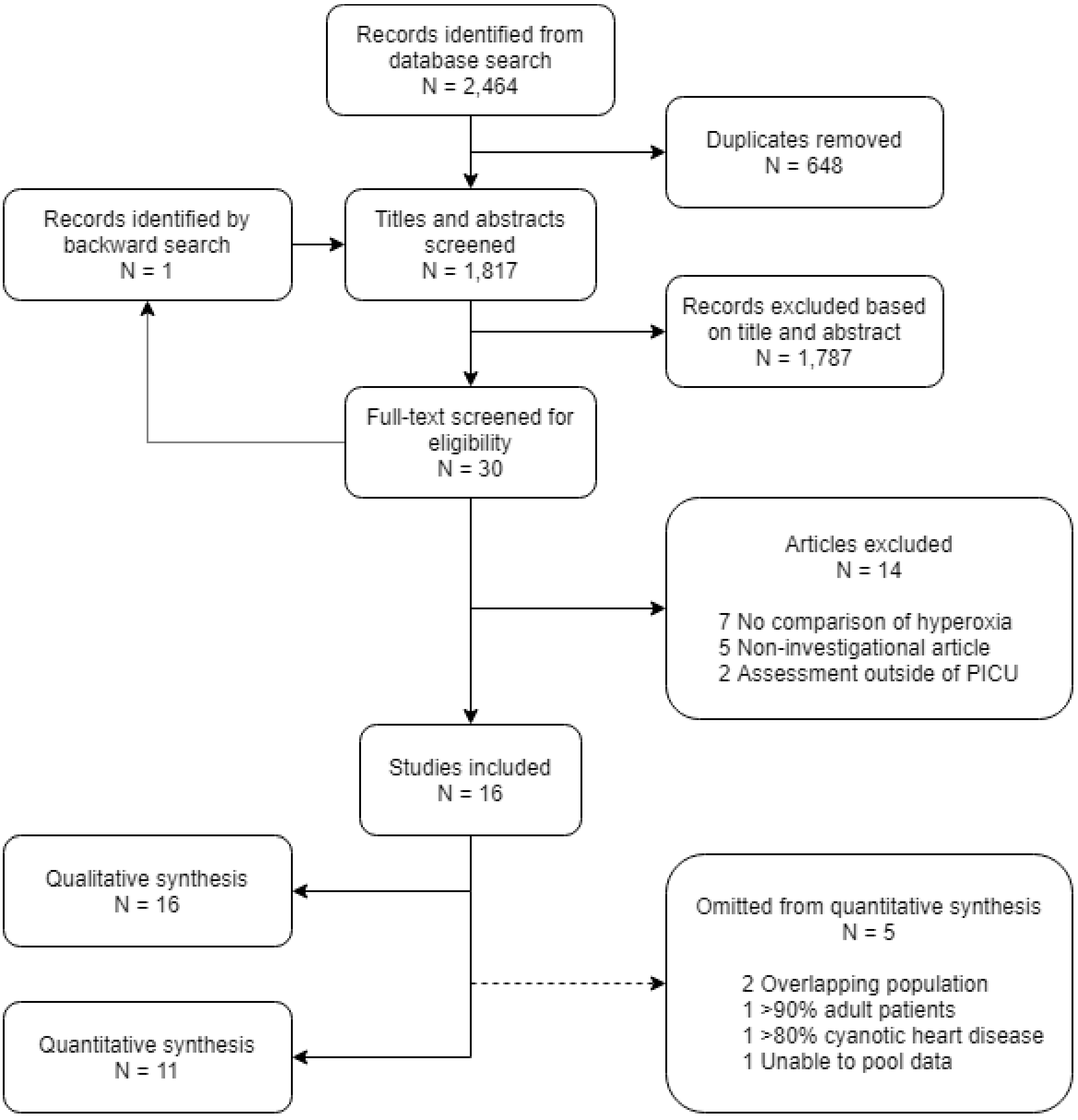

© 2022 Lilien TA et al. *JAMA Network Open.*

eFigure 2. Forest Plot of Hyperoxia (categorical) and Mortality, longest follow-up, stratified by Case mix

Study or Subgroup	log[Odds Ratio]	SE	Odds Ratio IV, Random, 95% CI
**1.4.1 Cardiac Arrest**			
Bennett 2013	0.1633	0.3038	1.18 [0.65, 2.14]
Del Castillo 2012	0.4822	0.4896	1.62 [0.62, 4.23]
Ferguson 2012	0.197	0.1518	1.22 [0.90, 1.64]
Guerra-Wallace 2013	-0.0576	0.5161	0.94 [0.34, 2.60]
López-Herce 2014	-0.1681	0.3973	0.85 [0.39, 1.84]
Van Zellem 2015	0.3287	0.2932	1.39 [0.78, 2.47]
**1.4.2 Traumatic Brain Injury**			
Ketharanathan 2020	0.5755	0.6292	1.78 [0.52, 6.10]
**1.4.3 Extracorporeal Membrane Oxygenation**			
Cashen 2018	0.8009	0.206	2.23 [1.49, 3.34]
Sznycer-Taub 2016	1.3464	0.5677	3.84 [1.26, 11.69]
**1.4.4 General**			
Kraft 2017	-0.1315	0.2689	0.88 [0.52, 1.49]
Numa 2018	1.2981	0.2966	3.66 [2.05, 6.55]
Pelletier 2020	-0.3773	0.1585	0.69 [0.50, 0.94]
Peters 2018	0	0	Not estimable
Raman 2016	0.5792	0.1445	1.78 [1.34, 2.37]
Ramgopal 2019	1.5162	0.1059	4.55 [3.70, 5.61]
Ramgopal 2020	0.9519	0.1333	2.59 [1.99, 3.36]

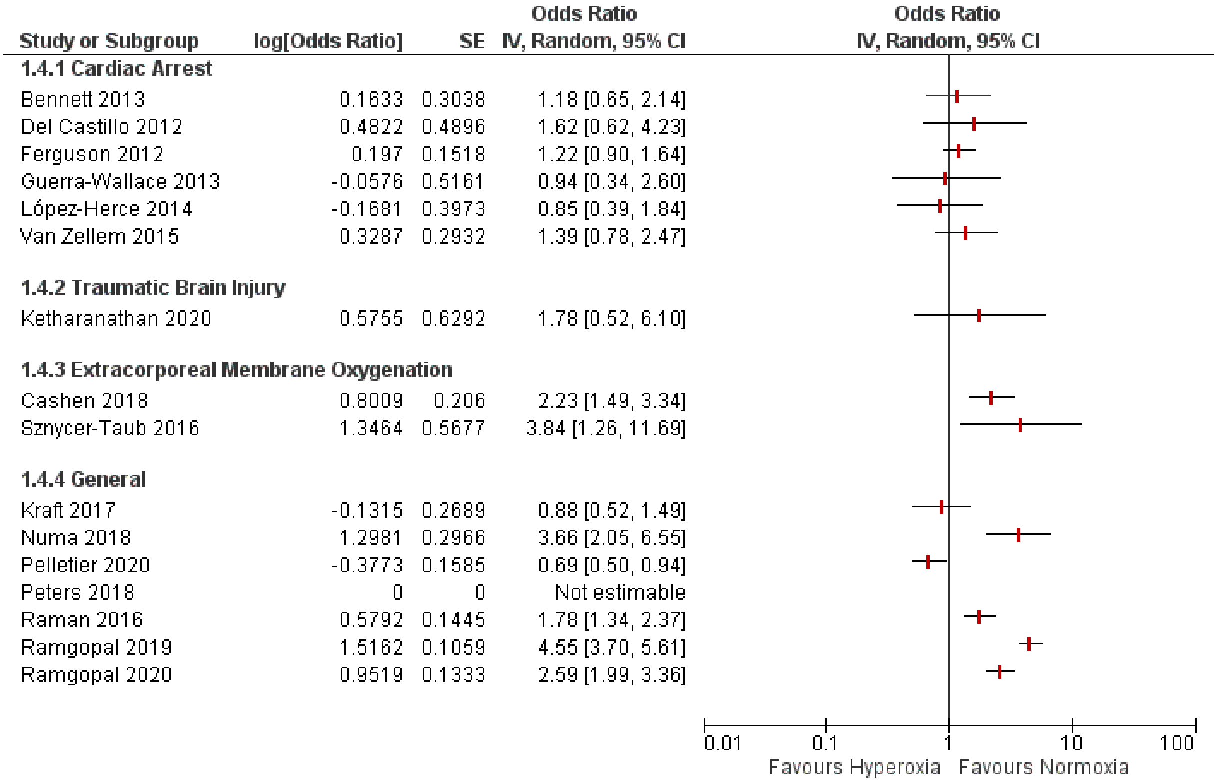

© 2022 Lilien TA et al. *JAMA Network Open.*

eFigure 3 Bajaut plot of the contribution to the overall heterogeneity and pooled effect estimate of each study

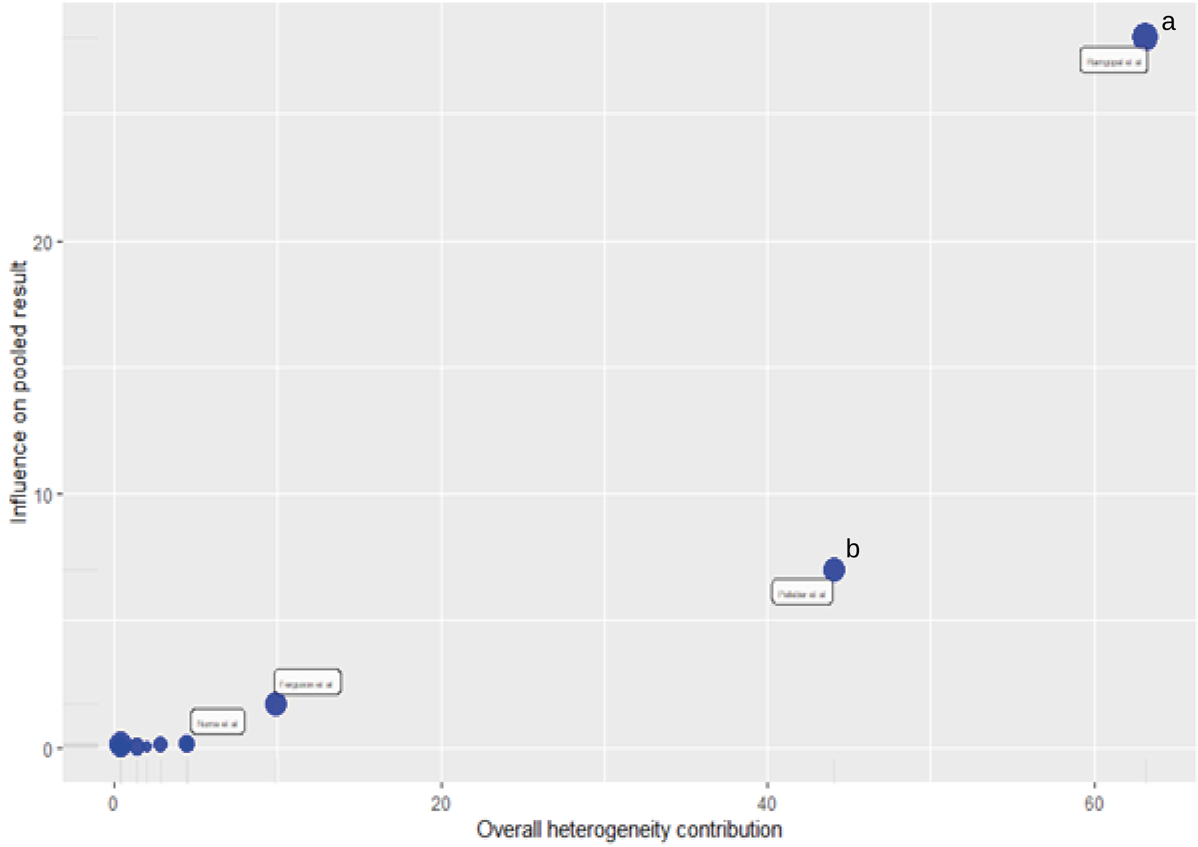

eFigure 3. a, Ramgopal et al.[14]; b, Pelletier et al.[10]

© 2022 Lilien TA et al. *JAMA Network Open.*

eFigure 4 Leave-One-Out plots sorted by the effect on overall heterogeneity and the pooled effect estimate

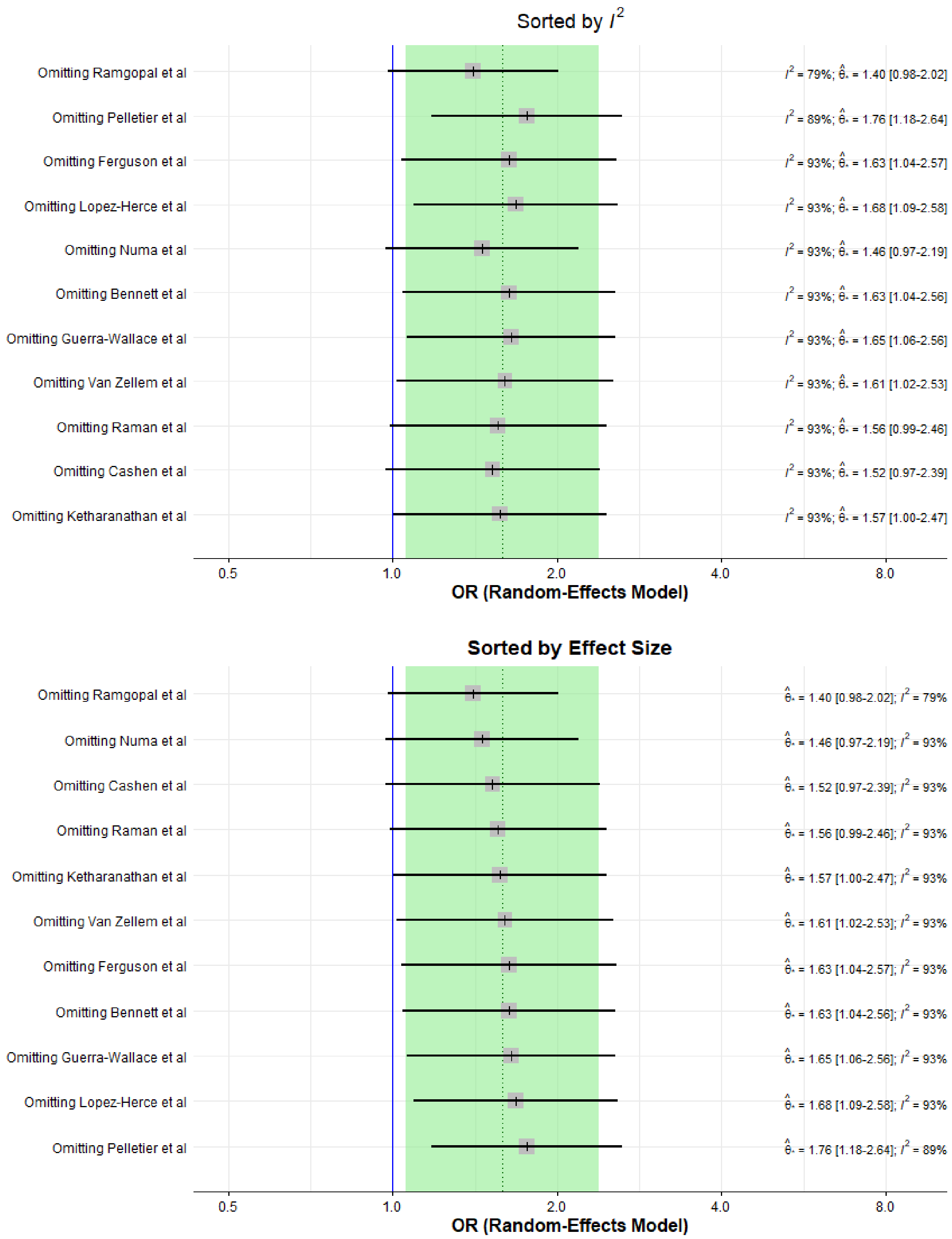

OR, odds ratio.

© 2022 Lilien TA et al. *JAMA Network Open.*

eFigure 5. GOSH plot of all subsets of studies with the overall heterogeneity and estimated pooled effect size and the three outlying studies based on the GOSH plot

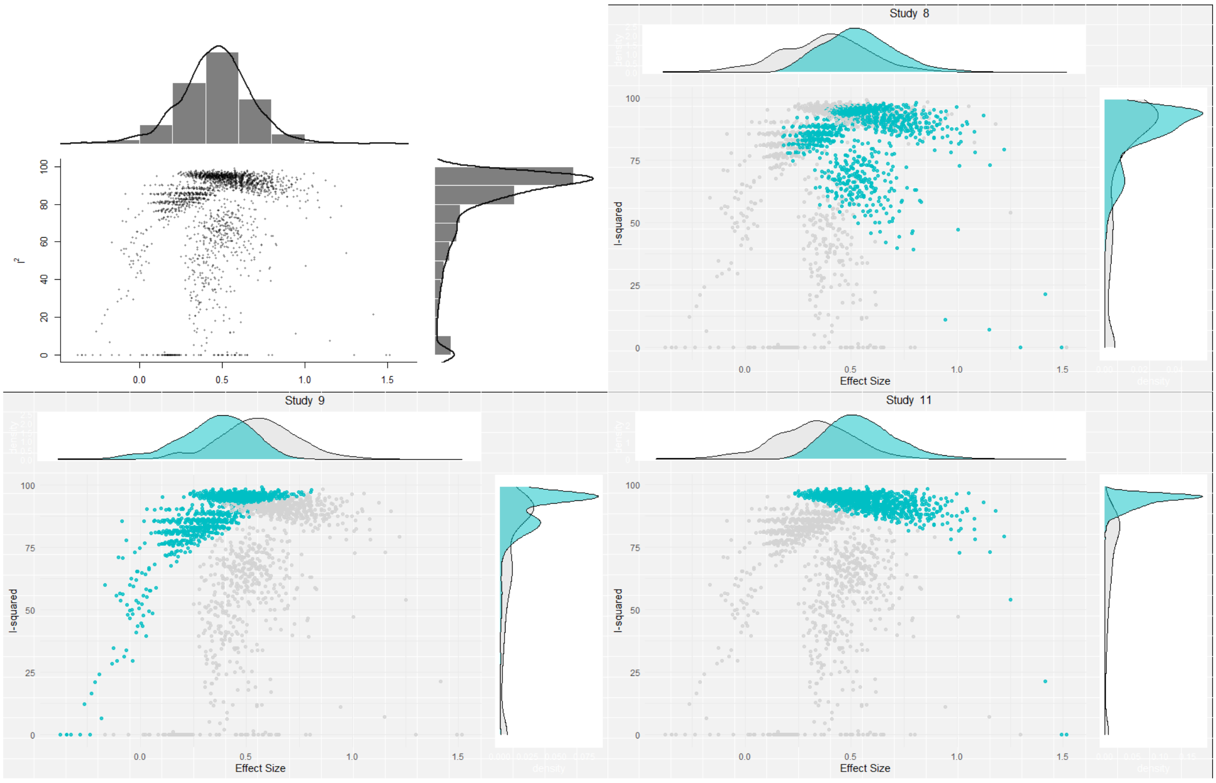

eFigure 5. Top left, GOSH-plot of all simulated subsets of studies; Study 8 (top right), Numa et al.[9]; Study 9 (bottom left), Pelletier et al[10]; Study 11 (bottom right), Ramgopal et al.[13]; GOSH, Graphic Display of Heterogeneity.

© 2022 Lilien TA et al. *JAMA Network Open.*

eFigure 6. Sensitivity analysis 1: excluding pronounced outliers

Study or Subgroup	log[Odds Ratio]	SE	Weight	Odds Ratio IV, Random, 95% CI
**1.6.1 Cardiac Arrest**				
Bennett 2013	0.1633	0.3038	10.7%	1.18 [0.65, 2.14]
Ferguson 2012	0.197	0.1518	17.5%	1.22 [0.90, 1.64]
Guerra-Wallace 2013	-0.0576	0.5161	5.4%	0.94 [0.34, 2.60]
López-Herce 2014	-0.1681	0.3973	7.8%	0.85 [0.39, 1.84]
Van Zellem 2015	0.3287	0.2932	11.1%	1.39 [0.78, 2.47]
**Subtotal (95% CI)**			**52.4%**	**1.18 [0.95, 1.48]**
Heterogeneity: Tau² = 0.00; Chi² = 1.24, df = 4 (P = 0.87); I² = 0%				
Test for overall effect: Z = 1.48 (P = 0.14)				
**1.6.2 Traumatic Brain Injury**				
Ketharanathan 2020	0.5755	0.6292	3.9%	1.78 [0.52, 6.10]
**Subtotal (95% CI)**			**3.9%**	**1.78 [0.52, 6.10]**
Heterogeneity: Not applicable				
Test for overall effect: Z = 0.91 (P = 0.36)				
**1.6.3 Extracorporeal Membrane Oxygenation**				
Cashen 2018	0.8009	0.206	14.8%	2.23 [1.49, 3.34]
**Subtotal (95% CI)**			**14.8%**	**2.23 [1.49, 3.34]**
Heterogeneity: Not applicable				
Test for overall effect: Z = 3.89 (P = 0.0001)				
**1.6.4 General**				
Numa 2018	1.2981	0.2966	10.9%	3.66 [2.05, 6.55]
Raman 2016	0.5792	0.1445	17.8%	1.78 [1.34, 2.37]
**Subtotal (95% CI)**			**28.8%**	**2.44 [1.21, 4.91]**
Heterogeneity: Tau² = 0.20; Chi² = 4.75, df = 1 (P = 0.03); I² = 79%				
Test for overall effect: Z = 2.50 (P = 0.01)				
**Total (95% CI)**			**100.0%**	**1.58 [1.21, 2.07]**
Heterogeneity: Tau² = 0.09; Chi² = 19.08, df = 8 (P = 0.01); I² = 58%				
Test for overall effect: Z = 3.33 (P = 0.0009)				
Test for subgroup differences: Chi² = 9.68, df = 3 (P = 0.02), I² = 69.0%				

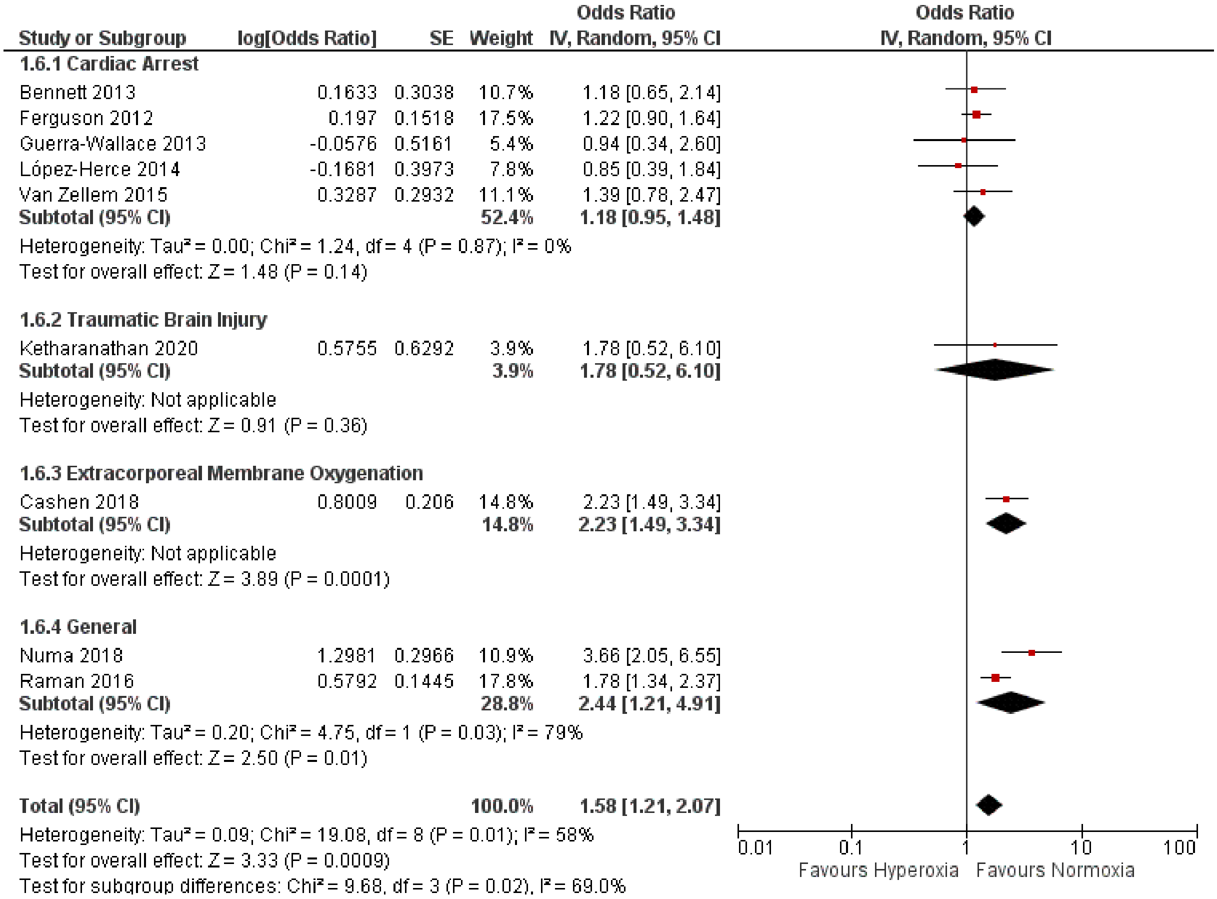

© 2022 Lilien TA et al. *JAMA Network Open.*

eFigure 7. Sensitivity analysis 2: excluding pronounced and potential outliers

Study or Subgroup	log[Odds Ratio]	SE	Weight	Odds Ratio IV, Random, 95% CI
**1.7.1 Cardiac Arrest**				
Bennett 2013	0.1633	0.3038	10.3%	1.18 [0.65, 2.14]
Ferguson 2012	0.197	0.1518	23.3%	1.22 [0.90, 1.64]
Guerra-Wallace 2013	-0.0576	0.5161	4.3%	0.94 [0.34, 2.60]
López-Herce 2014	-0.1681	0.3973	6.7%	0.85 [0.39, 1.84]
Van Zellem 2015	0.3287	0.2932	10.9%	1.39 [0.78, 2.47]
**Subtotal (95% CI)**			**55.5%**	**1.18 [0.95, 1.48]**
Heterogeneity: Tau² = 0.00; Chi² = 1.24, df = 4 (P = 0.87); I² = 0%				
Test for overall effect: Z = 1.48 (P = 0.14)				
**1.7.2 Traumatic Brain Injury**				
Ketharanathan 2020	0.5755	0.6292	3.0%	1.78 [0.52, 6.10]
**Subtotal (95% CI)**			**3.0%**	**1.78 [0.52, 6.10]**
Heterogeneity: Not applicable				
Test for overall effect: Z = 0.91 (P = 0.36)				
**1.7.3 Extracorporeal Membrane Oxygenation**				
Cashen 2018	0.8009	0.206	17.2%	2.23 [1.49, 3.34]
**Subtotal (95% CI)**			**17.2%**	**2.23 [1.49, 3.34]**
Heterogeneity: Not applicable				
Test for overall effect: Z = 3.89 (P = 0.0001)				
**1.7.4 General**				
Raman 2016	0.5792	0.1445	24.3%	1.78 [1.34, 2.37]
**Subtotal (95% CI)**			**24.3%**	**1.78 [1.34, 2.37]**
Heterogeneity: Not applicable				
Test for overall effect: Z = 4.01 (P < 0.0001)				
**Total (95% CI)**			**100.0%**	**1.46 [1.17, 1.83]**
Heterogeneity: Tau² = 0.03; Chi² = 10.68, df = 7 (P = 0.15); I² = 34%				
Test for overall effect: Z = 3.37 (P = 0.0008)				
Test for subgroup differences: Chi² = 9.44, df = 3 (P = 0.02), I² = 68.2%				

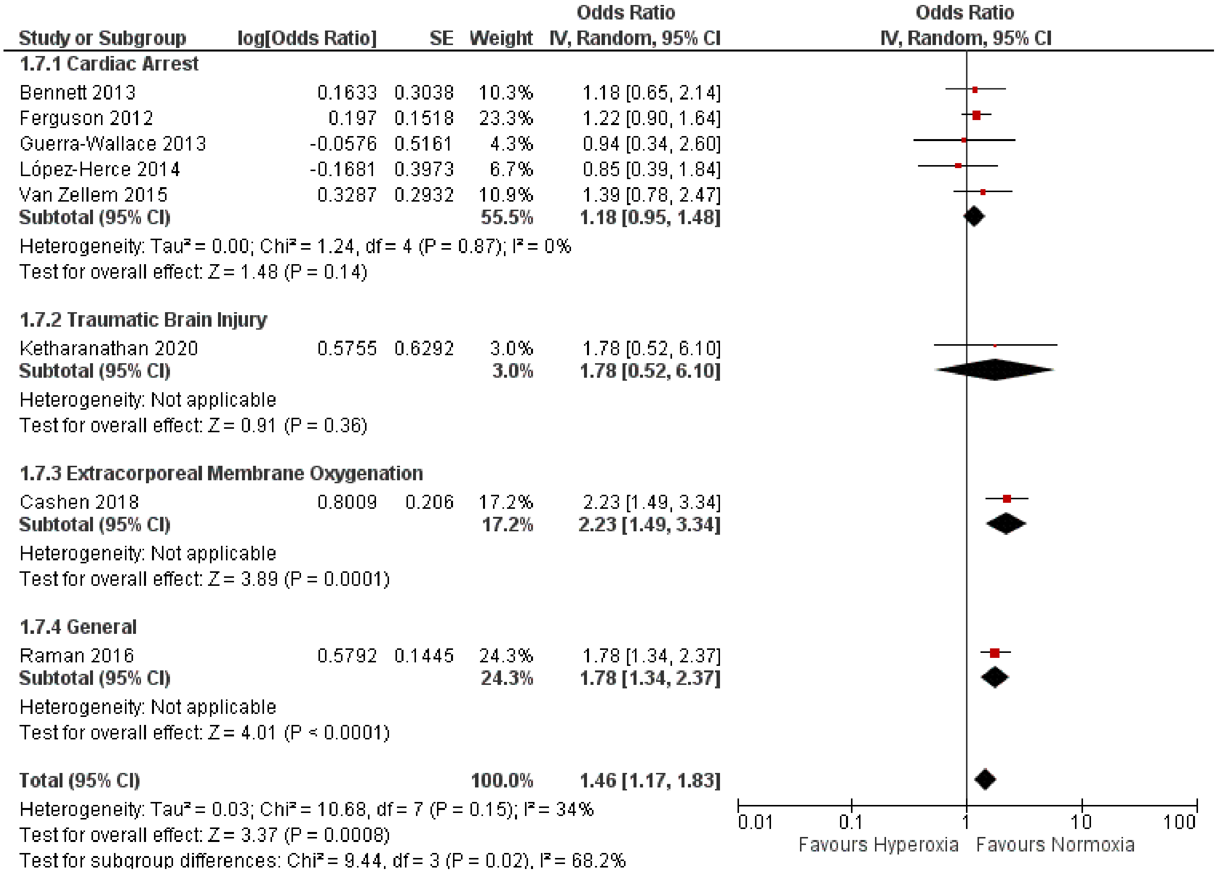

© 2022 Lilien TA et al. *JAMA Network Open.*

eFigure 8. Sensitivity analysis 3: excluding studies with only patients on ECMO support

Study or Subgroup	log[Odds Ratio]	SE	Weight	Odds Ratio IV, Random, 95% CI
**1.8.1 Cardiac Arrest**				
Bennett 2013	0.1633	0.3038	10.1%	1.18 [0.65, 2.14]
Ferguson 2012	0.197	0.1518	11.3%	1.22 [0.90, 1.64]
Guerra-Wallace 2013	-0.0576	0.5161	8.0%	0.94 [0.34, 2.60]
López-Herce 2014	-0.1681	0.3973	9.2%	0.85 [0.39, 1.84]
Van Zellem 2015	0.3287	0.2932	10.2%	1.39 [0.78, 2.47]
**Subtotal (95% CI)**			**48.8%**	**1.18 [0.95, 1.48]**
Heterogeneity: Tau² = 0.00; Chi² = 1.24, df = 4 (P = 0.87); I² = 0%				
Test for overall effect: Z = 1.48 (P = 0.14)				
**1.8.2 Traumatic Brain Injury**				
Ketharanathan 2020	0.5755	0.6292	7.0%	1.78 [0.52, 6.10]
**Subtotal (95% CI)**			**7.0%**	**1.78 [0.52, 6.10]**
Heterogeneity: Not applicable				
Test for overall effect: Z = 0.91 (P = 0.36)				
**1.8.4 General**				
Numa 2018	1.2981	0.2966	10.2%	3.66 [2.05, 6.55]
Pelletier 2020	-0.3773	0.1585	11.2%	0.69 [0.50, 0.94]
Raman 2016	0.5792	0.1445	11.3%	1.78 [1.34, 2.37]
Ramgopal 2019	1.5162	0.1059	11.5%	4.55 [3.70, 5.61]
**Subtotal (95% CI)**			**44.2%**	**2.11 [0.85, 5.23]**
Heterogeneity: Tau² = 0.82; Chi² = 105.29, df = 3 (P < 0.00001); I² = 97%				
Test for overall effect: Z = 1.62 (P = 0.11)				
**Total (95% CI)**			**100.0%**	**1.52 [0.92, 2.54]**
Heterogeneity: Tau² = 0.58; Chi² = 132.68, df = 9 (P < 0.00001); I² = 93%				
Test for overall effect: Z = 1.62 (P = 0.11)				
Test for subgroup differences: Chi² = 1.81, df = 2 (P = 0.40), I² = 0%				

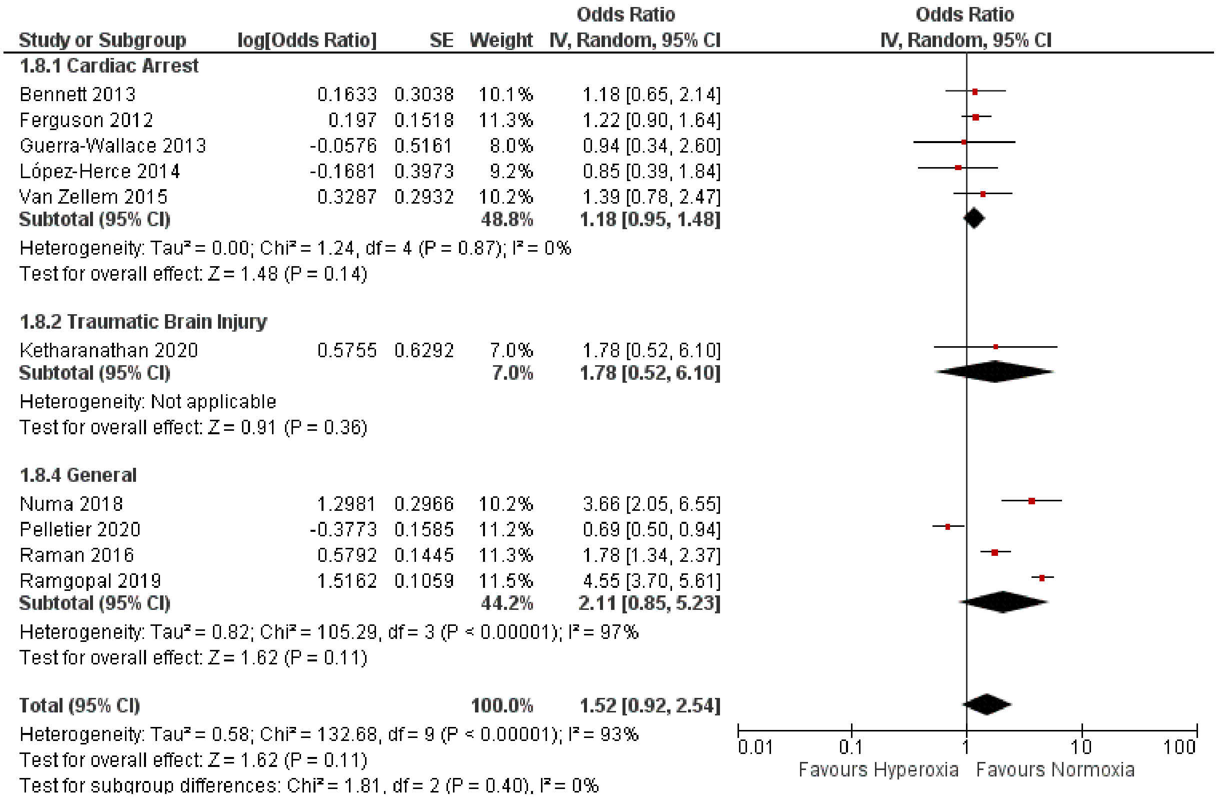

© 2022 Lilien TA et al. *JAMA Network Open.*

eFigure 9. Sensitivity analysis 4: including only studies with adjusted odds ratio for mortality

Study or Subgroup	log[Odds Ratio]	SE	Weight	Odds Ratio IV, Random, 95% CI
Numa 2018	0.9783	0.4925	21.3%	2.66 [1.01, 6.98]
Ramgopal 2019	0.5596	0.14	47.9%	1.75 [1.33, 2.30]
Van Zellem 2015	-0.2107	0.3437	30.8%	0.81 [0.41, 1.59]
**Total (95% CI)**			**100.0%**	**1.51 [0.85, 2.68]**

Heterogeneity: $Tau^2 = 0.16$; $Chi^2 = 5.38$, df = 2 (P = 0.07); $I^2 = 63\%$
Test for overall effect: Z = 1.41 (P = 0.16)

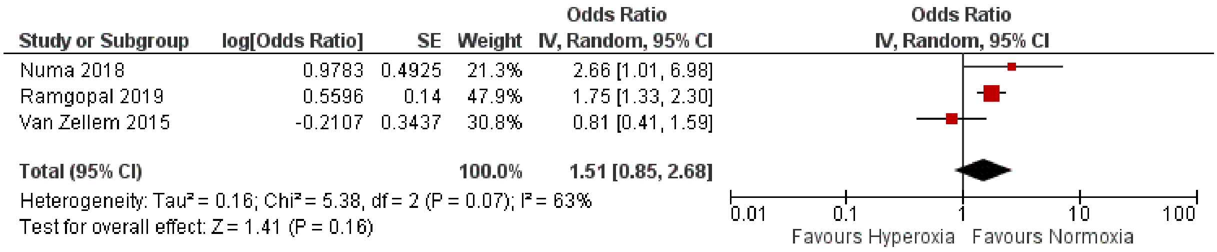

eFigure 10. Random-Effects Meta-analysis of Hyperoxia (Categorical) and Mortality, Longest Follow-up, Stratified by the Threshold of Hyperoxia With Exclusion of Pronounced Outlying Studies and Those Including Only Patients on ECMO Support (Subtotals)

Study or Subgroup	log[Odds Ratio]	SE	Weight	Odds Ratio IV, Random, 95% CI
**2.4.3 >200 mmHg (27 kPa)**				
Bennett 2013	0.1633	0.3038	23.2%	1.18 [0.65, 2.14]
Guerra-Wallace 2013	-0.4125	0.5907	7.0%	0.66 [0.21, 2.11]
Ketharanathan 2020	0.5828	0.8286	3.7%	1.79 [0.35, 9.09]
López-Herce 2014	-0.1681	0.3973	14.6%	0.85 [0.39, 1.84]
Numa 2018	0.7321	0.2736	27.4%	2.08 [1.22, 3.55]
Van Zellem 2015	0.2065	0.2968	24.1%	1.23 [0.69, 2.20]
**Subtotal (95% CI)**			**100.0%**	**1.29 [0.94, 1.77]**
Heterogeneity: Tau² = 0.02; Chi² = 5.72, df = 5 (P = 0.33); I² = 13%				
Test for overall effect: Z = 1.59 (P = 0.11)				
**2.4.4 >250 mmHg (33 kPa)**				
Ketharanathan 2020	1.0282	0.7102	14.7%	2.80 [0.70, 11.25]
Numa 2018	1.2981	0.2966	42.2%	3.66 [2.05, 6.55]
Van Zellem 2015	0.4812	0.2886	43.1%	1.62 [0.92, 2.85]
**Subtotal (95% CI)**			**100.0%**	**2.48 [1.36, 4.51]**
Heterogeneity: Tau² = 0.13; Chi² = 3.94, df = 2 (P = 0.14); I² = 49%				
Test for overall effect: Z = 2.96 (P = 0.003)				
**2.4.5 >300 mmHg (40 kPa)**				
Del Castillo 2012	0.4822	0.4896	8.9%	1.62 [0.62, 4.23]
Ferguson 2012	0.197	0.1518	23.4%	1.22 [0.90, 1.64]
Guerra-Wallace 2013	-0.0576	0.5161	8.3%	0.94 [0.34, 2.60]
Ketharanathan 2020	0.5755	0.6292	6.2%	1.78 [0.52, 6.10]
Numa 2018	1.5846	0.3469	13.5%	4.88 [2.47, 9.63]
Raman 2016	0.5792	0.1445	23.8%	1.78 [1.34, 2.37]
Van Zellem 2015	0.3287	0.2932	15.9%	1.39 [0.78, 2.47]
**Subtotal (95% CI)**			**100.0%**	**1.69 [1.19, 2.39]**
Heterogeneity: Tau² = 0.11; Chi² = 15.43, df = 6 (P = 0.02); I² = 61%				
Test for overall effect: Z = 2.97 (P = 0.003)				

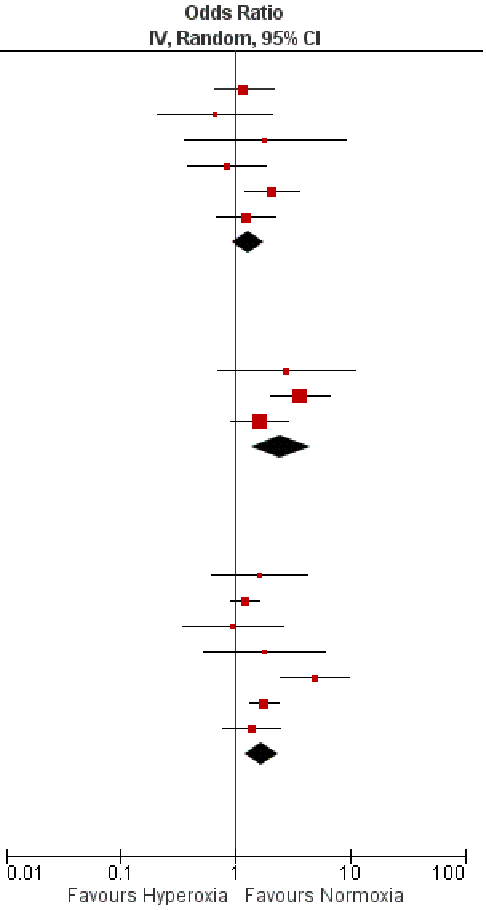

Test for subgroup differences: Chi² = 3.84, df = 2 (P = 0.15), I² = 47.9%

© 2022 Lilien TA et al. *JAMA Network Open.*

eFigure 11. Random-Effects Meta-analysis of Hyperoxia (Categorical) and Mortality, Longest Follow-up, Stratified by the Threshold of Hyperoxia With Exclusion of Pronounced and Potential Outlying Studies and Those Including Only Patients on ECMO Support (Subtotals)

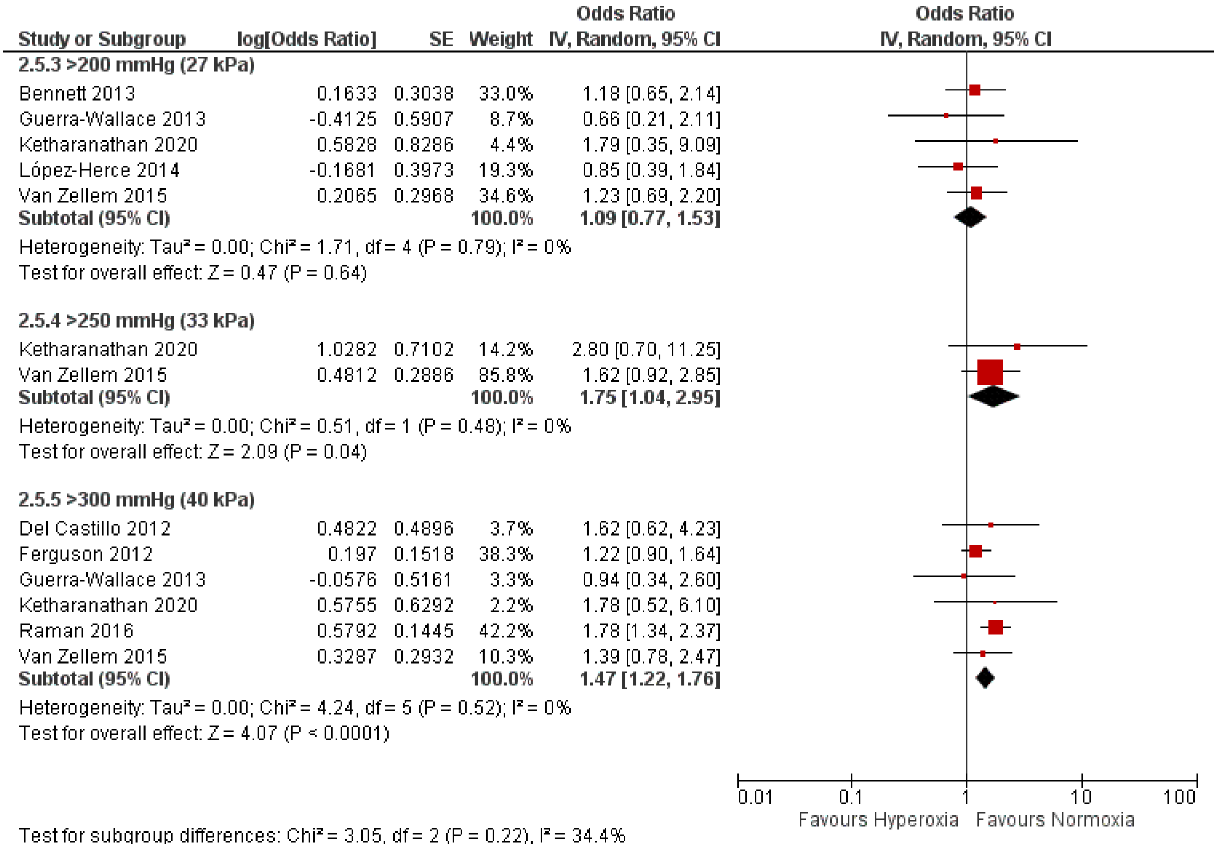

© 2022 Lilien TA et al. *JAMA Network Open.*

eFigure 12. Funnel plot of included studies in the main quantitative synthesis

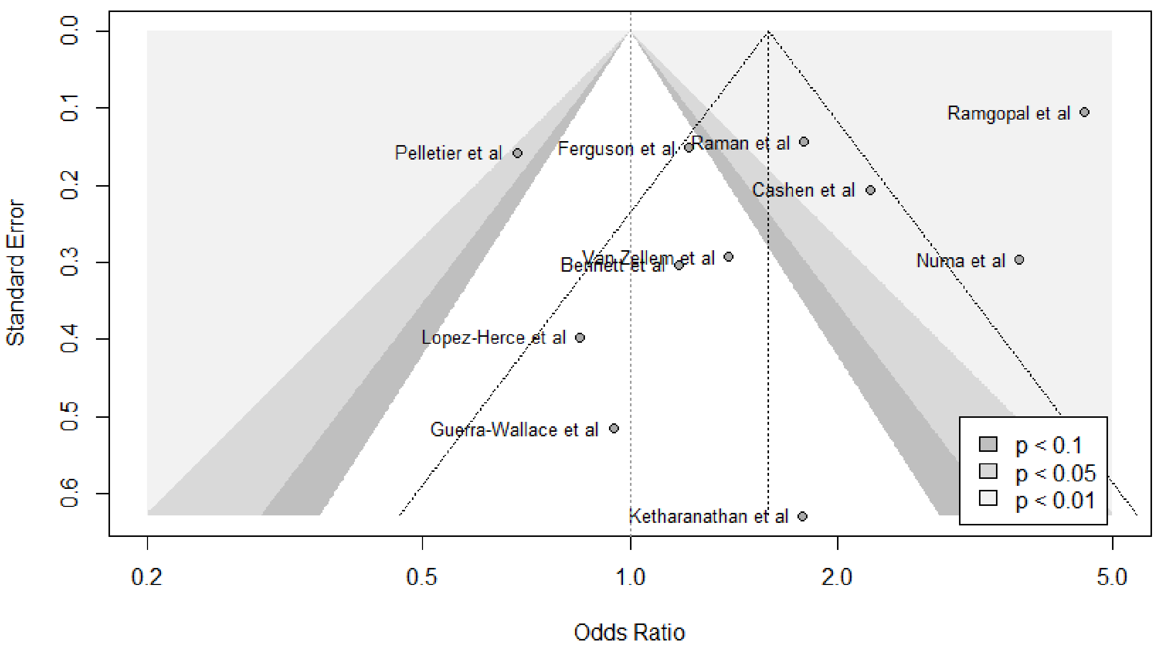

© 2022 Lilien TA et al. *JAMA Network Open.*

eReferences

1. Bennett KS, Clark AE, Meert KL, et al. Early oxygenation and ventilation measurements after pediatric cardiac arrest: Lack of association with outcome. Crit Care Med. 2013;41(6):1534-1542.

2. Cashen K, Reeder R, Dalton HJ, et al. Hyperoxia and hypocapnia during pediatric extracorporeal membrane oxygenation: Associations with complications, mortality, and functional status among survivors. Pediatr Crit Care Med. 2018;19(3):245-253.

3. del Castillo J, López-Herce J, Matamoros M, et al. Hyperoxia, hypocapnia and hypercapnia as outcome factors after cardiac arrest in children. *Resuscitation*. 2012;83(12):1456-1461.

4. Ferguson LP, Durward A, Tibby SM. Relationship between arterial partial oxygen pressure after resuscitation from cardiac arrest and mortality in children. Circulation. 2012;126(3):335-342.

5. Guerra-Wallace MM, Casey FL, Bell MJ, Fink EL, Hickey RW. Hyperoxia and hypoxia in children resuscitated from cardiac arrest. Pediatr Crit Care Med. 2013;14(3):e143.

6. Ketharanathan N, De Jonge RC., Klouwen I, et al. Hyperoxia in pediatric severe traumatic brain injury (TBI): a comparison of patient classification by cutoff versus cumulative (area-under-the-curve) analysis. Brain Inj. 2020;34(7):958-964.

7. Kraft F, Andel H, Gamper J, Markstaller K, Ullrich R, Klein KU. Incidence of hyperoxia and related in-hospital mortality in critically ill patients: a retrospective data analysis. Acta Anaesthesiol Scand. 2018;62(3):347-356.

8. Lopez-Herce J, Del Castillo J, Matamoros M, et al. Post return of spontaneous circulation factors associated with mortality in pediatric in-hospital cardiac arrest: a prospective multicenter multinational observational study. Crit Care. 2014;18(6):607.

9. Numa A, Aneja H, Awad J, et al. Admission Hyperoxia Is a Risk Factor for Mortality in Pediatric Intensive Care. Pediatr Crit Care Med. 2018;19(8):699-704.

10. Pelletier JH, Ramgopal S, Au AK, Clark RSB, Horvat CM. Maximum Pao 2 in the First 72 Hours of Intensive Care Is Associated With Risk-Adjusted Mortality in Pediatric Patients Undergoing Mechanical Ventilation. Crit Care Explor. 2020;2(9):e0186.

11. Peters MJ, Jones GAL, Wiley D, et al. Conservative versus liberal oxygenation targets in critically ill children: the randomised multiple-centre pilot Oxy-PICU trial. Intensive Care Med. 2018;44(8):1240-1248.

12. Raman S, Prince NJ, Hoskote A, Ray S, Peters MJ. Admission PaO2 and Mortality in Critically Ill Children. Pediatr Crit Care Med. 2016;17(10):e444-e450.

13. Ramgopal S, Dezfulian C, Hickey RW, et al. Association of Severe Hyperoxemia Events and Mortality Among Patients Admitted to a Pediatric Intensive Care Unit. JAMA Netw open. 2019;2(8):e199812.

14. Ramgopal S, Dezfulian C, Hickey RW, et al. Early hyperoxemia and outcome among critically ill children. Pediatr Crit Care Med. 2020;21(2):E129-E132.

15. Sznycer-Taub NR, Lowery R, Yu S, Owens ST, Hirsch-Romano JC, Owens GE. Hyperoxia Is Associated With Poor Outcomes in Pediatric Cardiac Patients Supported on Venoarterial Extracorporeal Membrane Oxygenation. Pediatr Crit Care Med. 2016;17(4):350-358.

16. van Zellem L, de Jonge R, van Rosmalen J, Reiss I, Tibboel D, Buysse C. High cumulative oxygen levels are associated with improved survival of children treated with mild therapeutic hypothermia after cardiac arrest. Resuscitation. 2015;90:150-157.

© 2022 Lilien TA et al. *JAMA Network Open.*
